# An Ultra-Stable, High-Energy and Wide-Temperature-Range Aqueous Alkaline Sodium-Ion Battery with the Microporous C_4_N/rGO Anode

**DOI:** 10.1007/s40820-024-01589-w

**Published:** 2025-02-24

**Authors:** Mengxiao Li, Rui Li, Huige Ma, Mingsheng Yang, Yujie Dai, HaiPing Yu, Yuxin Hao, Zhihui Wang, Bei Wang, Mingjun Hu, Jun Yang

**Affiliations:** 1 School of Materials Science and Engineering, Beihang University, Beijing, 100191 People’s Republic of China; 2 Beijing Institute of Nanoenergy and Nanosystems, Chinese Academy of Sciences, Beijing, 101400 People’s Republic of China; 3 School of Nanoscience and Engineering, University of Chinese Academy of Sciences, Beijing, 100049 People’s Republic of China; 4 ShenSi Lab, Shenzhen Institute for Advanced Study, University of Electronic Science and Technology of China, Shenzhen, 518110 People’s Republic of China

**Keywords:** Aqueous alkaline batteries, Organic anode, Ultra-high cycling stability, Alkaline antifreeze electrolyte, Wide temperature range

## Abstract

Common anode materials in aqueous alkaline electrolytes, such as cadmium, metal hydrides and zinc, usually suffer from remarkable biotoxicity, high cost, and serious side reactions. To overcome these problems, we develop a conjugated porous polymer (CPP) in-situ grown on reduced graphene oxide (rGO) and Ketjen black (KB), noted as C_4_N/rGO and C_4_N/KB respectively, as the alternative anodes. The results show that C_4_N/rGO electrode delivers a low redox potential (−0.905 V vs. Ag/AgCl), high specific capacity (268.8 mAh g^−1^ at 0.2 A g^−1^), ultra-stable and fast sodium ion storage behavior (216 mAh g^−1^ at 20 A g^−1^) in 2 M NaOH electrolyte. The assembled C_4_N/rGO//Ni(OH)_2_ full battery can cycle stably more than 38,000 cycles. Furthermore, by adding a small amount of antifreeze additive dimethyl sulfoxide (DMSO) to adjust the hydrogen bonding network, the low-temperature performance of the electrolyte (0.1 DMSO/2 M NaOH) is significantly improved while hydrogen evolution is inhibited. Consequently, the C_4_N/rGO//Ni(OH)_2_ full cell exhibits an energy density of 147.3 Wh Kg^−1^ and ultra-high cycling stability over a wide temperature range from −70 to 45 °C. This work provides an ultra-stable high-capacity CPP-based anode and antifreeze electrolyte for aqueous alkaline batteries and will facilitate their practical applications under extreme conditions.

**Supplementary Information:**

The online version contains supplementary material available at 10.1007/s40820-024-01589-w.

## Highlights

- An integrated conjugated microporous polymer composite electrode (C_4_N/rGO) with high conductivity, large specific surface area and good solvent resistance was prepared by in-situ growth.
- An antifreeze alkaline electrolyte (0.1 DMSO/2 M NaOH) was developed to broaden the operation temperature zone and voltage window of the aqueous alkaline battery.
- The prepared aqueous alkaline battery exhibits a high energy density (147.3 Wh Kg^−1^ at 25 °C), outstanding long cycling stability and excellent wide-temperature-range performance (−70 to 45 °C).

**Supplementary Information:**

The online version contains supplementary material available at 10.1007/s40820-024-01589-w.

## Graphical abstract

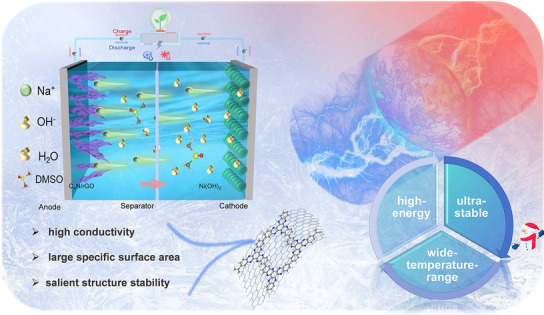

## Introduction

In recent years, rechargeable batteries have received widespread attention as an efficient energy storage system to meet the development needs of a green economy in society [1]. Sodium is 440 times more abundant than lithium in the Earth's crust and is widely distributed and simple to extract [2]. Considering the abundance of the element and the cost of the resource, sodium has appeared as a substitute for lithium in recent years and has gained more and more attention in the field of batteries [3–6]. Aqueous rechargeable batteries have low cost, high safety and reliability, and fast kinetics compared with the organic electrolyte-based batteries, which meet the needs of modern green economy development [7, 8]. Therefore, the interest in aqueous rechargeable batteries is increasing day by day.

Conventional electrolytes for aqueous rechargeable batteries are commonly H_2_SO_4_ for acidic batteries [9], alkali metal salts (Li^+^, Na^+^) for neutral batteries [10], and MOH (M = alkali metal) for alkaline batteries [11]. Aqueous alkaline batteries are the latent high energy rechargeable batteries with prospects for large-scale energy storage applications [12]. Commercial aqueous alkaline batteries usually used nickel-based cathode (Ni(OH)_2_, theoretical specific capacity 289 mAh g^−1^) and metal or alloy anodes (zinc, iron, cadmium, hydrogen storage alloys, etc.) [13]. In 2022, Zhou et al. reviewed nickel-based rechargeable aqueous alkaline batteries such as Ni–Fe, Ni–Cd, Ni–MH, and Ni–Zn, and recounted their advantages and operating principles [14]. Since the anode materials of aqueous alkaline batteries always have some shortcomings [11], it is the quest of researchers to find anode materials with low cost, environmental friendliness, high specific capacity and suitable redox potentials [15–17].

Conjugated porous polymers (CPPs) [18] are constructed by strong covalent bonds and can be designed to contain a lot of reactive groups. Their porous structure and large π-conjugated system facilitate rapid charge transport and collection of electrons and ions during charge/discharge processes [19], meeting most of the requirements for electrode materials, but the conductivity of such materials is still unsatisfactory [20]. Current studies often use conductive carbon such as Ketjen Black (KB) [21] and reduced graphene oxide (rGO) to compound with organics and mitigate their poor conductivity. The integration of two-dimensional (2D) graphene [22] layers with covalent organic frameworks (COFs) can further stabilize the structure and provide an interconnected conductive network for electron transfer, leading to electrode materials with enhanced electrochemical properties [23]. In addition, with the extension of battery application scenarios [24], the demand for special applications, such as daily use in cold regions, aerospace, and polar exploration, etc., becomes more urgent [25], and aqueous alkaline batteries need to have better environmental adaptability [26–28]. It has been reported that dimethyl sulfoxide (DMSO) has good polarity and stability in both acidic and alkaline media, and can dissolve a variety of inorganic salts and inhibit hydrogen evolution, showing good compatibility with aqueous alkaline electrolyte [29]. However, DMSO, as a universal organic solvent [30], can dissolve most organic small molecules, apt to cause the dissolution of organic electrode. Thus, the electrolyte in the presence of DMSO has a higher demand for the stability of electrode materials [31].

Herein, we prepared a C_4_N/rGO anode material by a solvothermal method where a conjugated porous organic polymer C_4_N was in-situ grown on rGO [32]. The effect of different conductive carbon templates (KB and rGO) on the electrochemical properties of C_4_N was investigated in 2 M NaOH, and the results show that the C_4_N/rGO electrode has more excellent electrochemical properties in terms of specific capacity (268.8 mAh g^−1^ at 0.2 A g^−1^) and cycling stability (84.2% after 2000 cycles at 1 A g^−1^), which is attributed to the high conductivity (1.057 × 10^2^ S m^−1^), large specific surface area, and salient structure stability of in-situ synthesized C_4_N/rGO composite electrodes. The assembled C_4_N/rGO//Ni(OH)_2_ full cell presents a high energy density (134 Wh Kg^−1^) and superior cycling stability (85.5% after 38,000 cycles at 10 A g^−1^). By adding a small amount of antifreeze additive DMSO to 2 M NaOH electrolyte, the antifreeze ability of the aqueous alkaline electrolyte was significantly improved and the hydrogen evolution side reaction was inhibited. The developed C_4_N/rGO//Ni(OH)_2_ full batteries exhibit excellent battery performance at ultra-low temperatures, as shown in the following: at −40 °C, the battery capacity is still 92% of that at room temperature, and the capacity retention rate is 114% at 10 A g^−1^ over 12,500 cycles; at −70 °C, the battery can cycle stably for 160 cycles with a capacity retention of 91%. The aqueous alkaline battery based on C_4_N/rGO anode and 0.1 DMSO/2 M NaOH electrolyte owns low cost, high safety, high energy density (147.3 Wh Kg^−1^ at 25 °C), long-term cycle stability and good adaptability over a wide temperature range (−70 to 45 °C), and offers a new option for the development of high-performance wide-temperature-range aqueous battery.

## Experimental Section

### Chemicals

Triquinoyl Hydrate (98%) and 3,3′,4,4′-tetraaminobiphenyl (99%) were purchased from Macklin. reduced graphene oxide (rGO) was prepared by calcining graphene oxide in an argon tube furnace at 800 °C for 1 h, where graphene oxide was prepared from natural graphite powder (99.95%, Macklin) using an improved Hummers method. KB was purchased from Guangdong Canrd New Energy Technology Co. Ltd. (Dongguan, China). NaOH (98%) and sulphuric acid (98%) were purchased from TCI Chemicals. N-methyl-2-pyrrolidone (99%) and methanol (99.5%) were purchased from Aladdin. Dimethyl sulfoxide (DMSO) was purchased from JSENB.

### Fabrication of Electrodes

#### Preparation of C_4_N, C_4_N/KB_0.3_, C_4_N/KB_0.45_, and C_4_N/rGO_0.45_ Anodes

Triquinoyl Hydrate (HKH, 0.138 g), N-methyl-2-pyrrolidone (NMP, 25 mL), 3,3′,4,4′-tetraaminobiphenyl (BPTA, 0.140 g) and methanol (8 mL) were added to a pressure-resistant tube. The pressure-resistant tube was then placed at a low temperature for a period of time, removed and some H_2_SO_4_ was added as a catalyst, stirred well, and then the reaction system was allowed to react for two days at 175 °C under argon gas environment. The resultant product was filtered and washed several times with ethanol, water and methanol in the process. The sample was then dried in a vacuum oven at 100 °C for 24 h. Finally, 0.21 g of reddish-brown powder, denoted as C_4_N, was obtained. The yield is 75.5%.

In the above reaction, with other reaction conditions kept constant, 0.105 and 0.210 g of KB were added to the pressure-resistant tube and the resulting products were noted as C_4_N/KB_0.3_ (0.317 g), and C_4_N/KB_0.45_ (0.424 g), respectively. Similarly, 0.210 g of rGO was added, and the resulting black powder sample was labelled as C_4_N/rGO_0.45_ (0.421 g).

The C_4_N electrode sheets were prepared by homogeneously mixing the C_4_N materials with KB and polyvinylidene fluoride (PVDF) in NMP in the weight ratio of 6:3:1, then grinding them into a slurry, uniformly coating the slurry on carbon paper and drying it for 12 h in a vacuum oven at 120 °C. The other three electrode sheets (C_4_N/KB_0.3_, C_4_N/KB_0.45_, and C_4_N/rGO_0.45_) were prepared by mixing the organic materials with the PVDF binder in NMP at a ratio of 9:1 to form the slurries, and the other steps were the same as the above. Finally, the corresponding anodes were obtained. The loading amount of the active substance (C_4_N) is about 0.88–1 mg cm^−2^.

#### Preparation of Ni(OH)_2_ Cathode

According to the weight ratio of β-Ni(OH)_2_: Ni powder: PVDF = 6:3:1, the mixture was milled inside the NMP to form a slurry, and then the slurry was uniformly coated on the nickel mesh and then dried in a vacuum oven at 120 °C for 24 h. The cathode was obtained.

### Preparation of Electrolytes

Firstly, solutions with different DMSO molar fraction ratios (DMSO/(DMSO + H_2_O)) of 0, 0.01, 0.1, and 0.2 were configured, and then an appropriate amount of NaOH was added to make the concentration of NaOH to be 2 M. Stirring was done to make it fully dissolved, and finally, the desired electrolyte was obtained.

### Characterizations

Scanning electron microscopy (SEM) (ZEISS GeminiSEM 300) and transmission electron microscopy (TEM) (Tecnai G2 F20 S-TWIN TMP) were used to observe the sample morphology as well as for elemental analysis. Lattice spacing was measured by high resolution TEM (HRTEM). Fourier transform infrared spectroscopy (FTIR) was obtained on a Thermo Scientific Nicolet iS50 instrument. X-ray photoelectron spectroscopy (XPS) spectra were obtained by Thermo Scientific K-Alpha. Organic elemental analysis (EA) was obtained by Thermo Scientific FlashSmart instrument. X-ray diffraction (XRD) was obtained by the Rigaku SmartLab SE instrument. The Specific surface area and pore size of the materials were analyzed by Brunauer–Emmett–Teller (BET) (Micromeritics 3Flex), and the pore size was analysed by full pore analysis using the density functional theory (DFT) model. The electronic conductivity of the samples was tested by a conductivity tester. DSC was performed by Netzsch DSC 200 F3. Raman spectra were obtained by the Horiba LabRAM HR Evolution test.

### Electrochemical Measurements

C_4_N, C_4_N/KB_0.3_, C_4_N/KB_0.45_ and C_4_N/rGO_0.45_ anode materials were used as working electrodes, activated carbon was used as a counter electrode, Ag/AgCl was used as reference electrode, and the electrolyte was 2 M NaOH to form a half-cell, which was tested using Swagelok cells. In the full cell, C_4_N/rGO_0.45_ was used as the anode material, β-Ni(OH)_2_ was used as the cathode material, and the electrolyte was 2 M NaOH with different molar fractions of DMSO added (DMSO/2 M NaOH). The full cell was tested using a button cell, where the mass ratio of the cathode to anode was approximately 1.5:1. Electrochemical performance tests (linear scanning voltammetry (LSV), cyclic voltammetry (CV) and electrochemical impedance spectroscopy (EIS)) were performed on an AMETEK Princeton Applied Research Electrochemical workstation. The ionic conductivity is calculated by $\upsigma = \frac{{\text{L}}}{{{\text{RS}}}}$, where L is the thickness of the glass fiber separator, R is the starting point of impedance spectra, and S is the area of the electrode sheet. Energy density is equal to the rated capacity of the battery (Ah) multiplied by the average battery operating voltage (V) and then divided by the mass of the battery (kg), where the mass of the battery is the total mass of the active substance C_4_N plus nickel hydroxide, and the battery operating voltage is the average discharge medium voltage. Galvanostatic charge/discharge (GCD) testing of the batteries was performed on a LANHE battery test system (CT3001A). Room temperature was controlled by a battery test incubator (BLC-300), low temperature by an ultra-low temperature refrigerated storage box (DW-HL340), and high temperature by a blast drying oven (101-0B).

### Computational Method

The DFT calculations were carried out under the B3LYP level and 6-31 +  + basis set with Gaussian 09 program. All the structures were first optimized until the maximum force converged into 0.00045 a.u, and later the molecular orbitals were retrieved from the output files of Gaussian 09 program. The classical molecular dynamics (MD) simulations were carried out using GROMACS. The AMBER-99 force field [33] was used along with Restrained Electro Static Potential (RESP) charge generated by Multiwfn [34]. Initially, 54 NaOH and 1,500 H_2_O molecules were packed into a 45 × 45 × 45 Å^3^ box using the packmol [35] to simulate the 2 M NaOH. For 2 M NaOH/0.1 DMSO system, 54 NaOH, 1042 water, and 116 DMSO molecules were packed. All the systems were first heated up to target temperatures (298.15 K) from 10 K and followed by 5 ns equilibration under isothermal-isobaric ensemble (NPT) at 1 bar. For the production run, another 10 ns NPT simulations were performed. For NPT simulations, the temperature was controlled by a Nosé-Hoover thermostat and the pressure was controlled using the C-rescale coupling. Electrostatic interactions were treated using the Particle-Mesh-Ewald (PME) method [36]. The hydrogen bond numbers were calculated by the MD Analysis program.

## Result and Discussion

### Synthesis and Characterization of Anode Materials

**Fig. 1:**
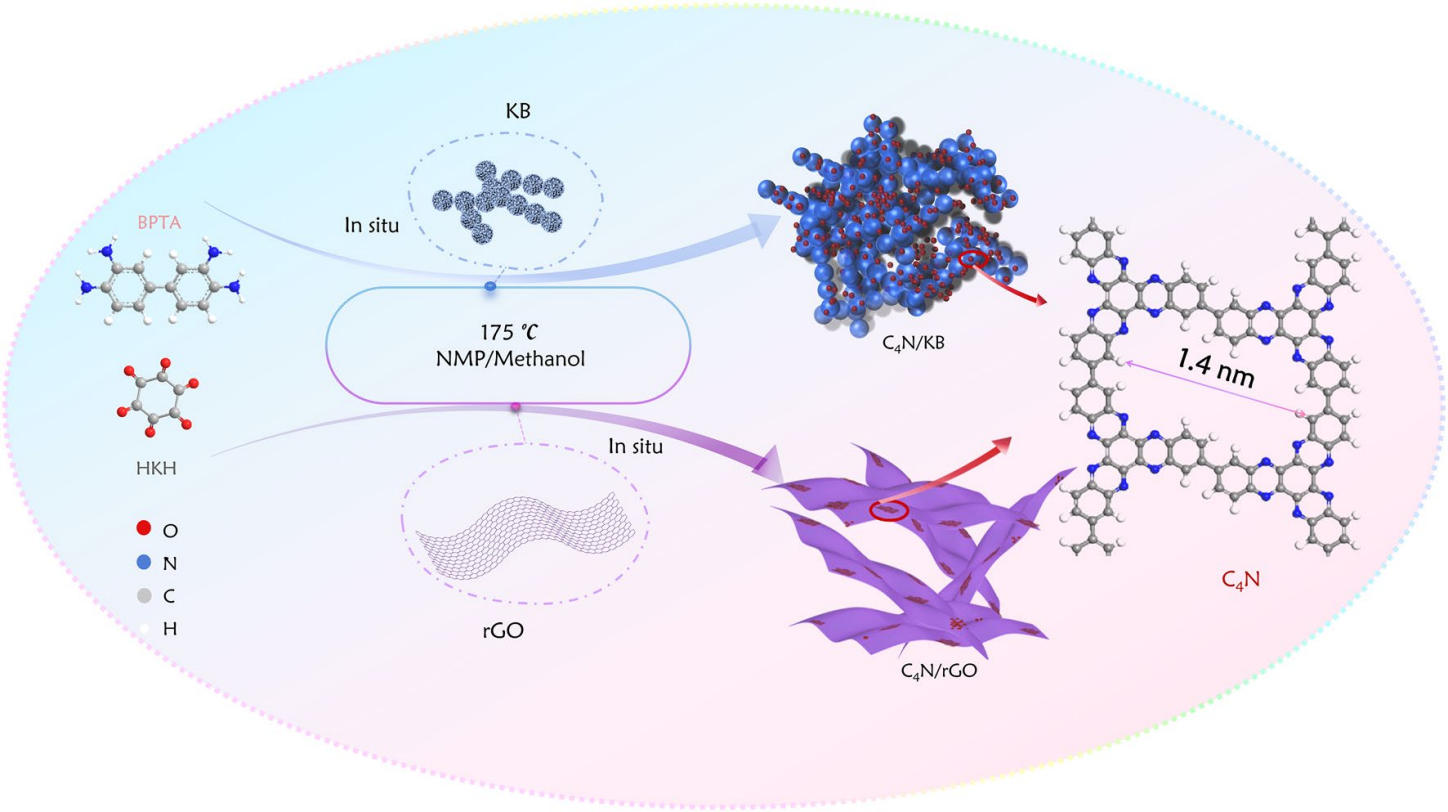
Schematic illustration of the preparation of anode materials C_4_N/KB, C_4_N/rGO

The schematic diagram (Fig.  1) depicts the synthesis routes of the C_4_N-based negative electrode materials. C_4_N was produced by a simple condensation reaction using BPTA and cyclohexanone octahydrate (HKH) as the precursors, and C_4_N/KB and C_4_N/rGO electrode materials were prepared by in-situ growth of C_4_N on KB or GO substrates under solvothermal conditions. The molecular structure of C_4_N is shown in Fig.  1, and the pore size is expected to be 1.4 nm based on the quantum chemical method and our previous report [37].

**Fig. 2:**
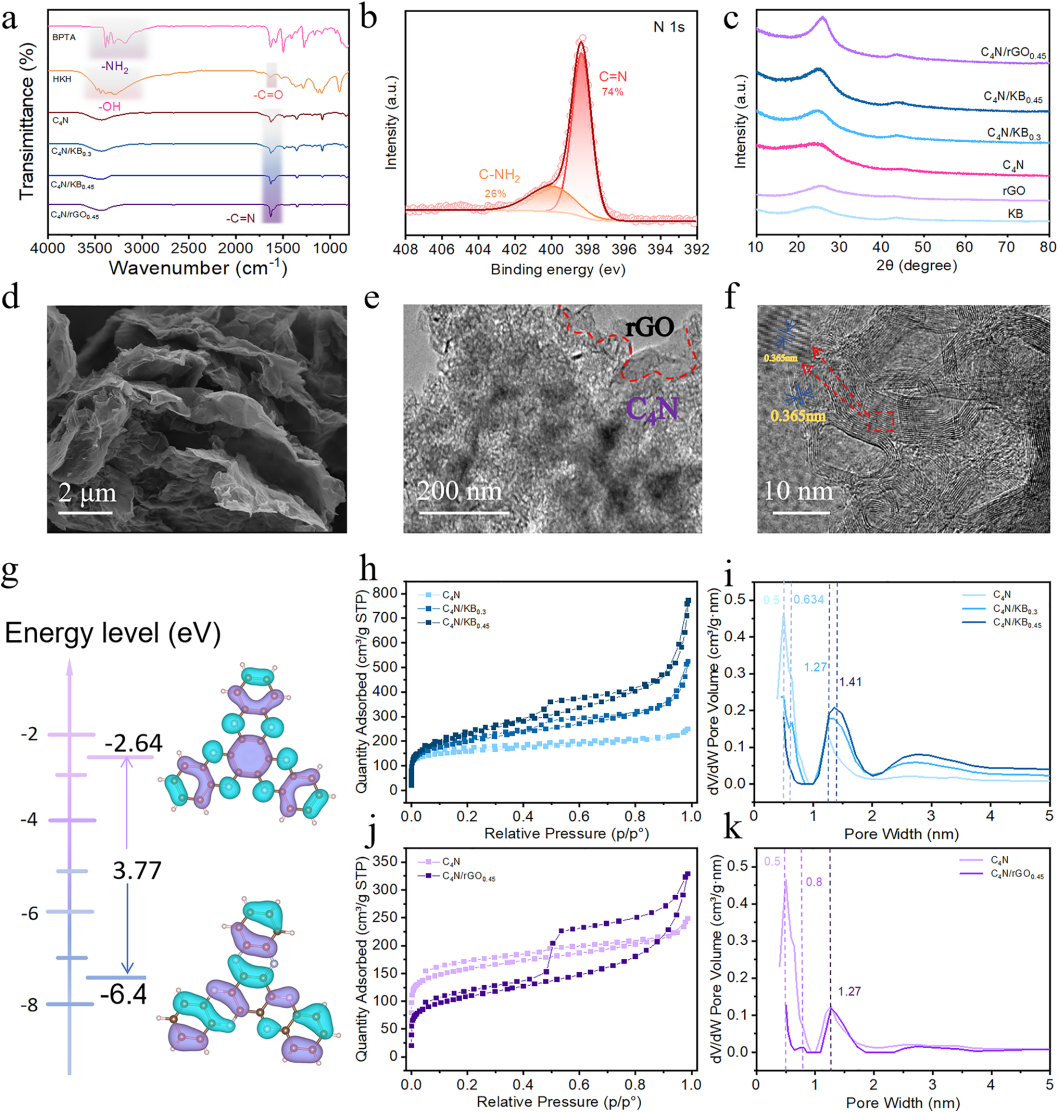
**a** FT-IR of the precursors BPTA, HKH and the products C_4_N, C_4_N/KB_0.3_, C_4_N/KB_0.45_ and C_4_N/rGO_0.45_. **b** High-resolution N 1*s* XPS spectra of C_4_N. **c** XRD of KB, rGO, C_4_N, C_4_N/KB_0.3_, C_4_N/KB_0.45_ and C_4_N/rGO_0.45_. **d** SEM, **e** TEM and **f** HRTEM images of C_4_N/rGO_0.45_. **g** LUMO/HOMO energy levels of C_4_N. **h** Specific surface area and **i** pore size of C_4_N, C_4_N/KB_0.3_ and C_4_N/KB_0.45_. **j** Specific surface area and **k** pore size of C_4_N and C_4_N/rGO_0.45_

FTIR spectra (Fig. 2a) tentatively confirmed the change in functional groups of the product, with an IR absorption peak of C=N at 1634 cm^−2^ appearing, and the broad peaks corresponding to –NH_2_ of BPTA at 3200–3400 cm^−1^ and the peaks corresponding to C=O groups of HKH at 1641 cm^−1^ disappearing compared to the precursors [38]. These results indicated that the ketamine condensation reaction between BPTA and HKH occurred. Organic elemental analysis results of the synthesized C_4_N material (Table S1) showed that the mass fractions of C and N were 62.76% and 18.25%, with the atomic ratio of C to N 4.01:1, well matched with the theoretical C/N ratio of 4:1, reconfirming the successful synthesis of C_4_N. The high-resolution N 1*s* XPS spectra of C_4_N (Fig. 2b) were fitted to two sub-peaks at 398.4 and 400.4 eV, corresponding to the C=N and C–NH_2_ bonds, respectively [39]. In addition, XRD (Fig. 2c) exhibited a broad diffraction peak at 25°, indicating that C_4_N had an amorphous structure [19]. The electrode materials after compounding with conductive carbon substrates were all characterized by the broad diffraction peaks of carbon materials corresponding to the (002) and (100) crystal planes of GO and KB, respectively. The broad XRD diffraction peaks around 25° were attributed to π-π stacking conjugated structure, which was favorable for electron transport and the ion diffusion in the electrode materials and promoted the reaction kinetics [23].

SEM images, energy dispersive spectrometer (EDS) mapping (Figs. S1 and S2) and TEM images (Figs. S3 and S4) showed that C_4_N particles were coated on the surface of KB particles, and the composite electrode material existed in the form of densely packed nanoparticles in which KB and C_4_N were interspersed and formed a homogeneous commixture. For C_4_N/rGO composites (Figs. 2d, e and S1e), the wrinkled and curved rGO nanosheet structure was maintained, and C_4_N was densely distributed in the form of nanoparticles on the surface of rGO. EDS shows that C and N elements are uniformly distributed over the surface of the sample. The FT-IR spectra and SEM–EDS mapping of KB and rGO were shown in Fig. S5. TEM shows that C_4_N exists in the form of interlinked nanoparticles with a large number of indeterminate carbon structures present at the periphery of the particles, and C_4_N particles show curved lattice fringes with an interplanar spacing of 0.365 nm corresponding to π-π stacking distance. Similarly, the crystal facets with a spacing of 0.365 nm were also observed in the HRTEM images of C_4_N/KB and C_4_N/rGO (Fig. 2f), corresponding to (002) crystal facets in XRD. Among the three samples, C_4_N grown in situ on rGO shows better crystallinity than C_4_N alone and C_4_N/KB, may attribute to the template effect of 2D rGOs. The energy gap of C_4_N was calculated based on the lowest unoccupied molecular orbital (LUMO) and highest occupied molecular orbital (HOMO) of HATN to be 3.77 eV (Fig. 2g). The conductivities (Table S2) of the materials were greatly enhanced by in situ compounding with the conductive additives, and were 4.174 × 10^–12^, 5.860 × 10^–5^, 5.228 × 10^–2^, and 1.057 × 10^2^ S m^−1^ respectively, for C_4_N, C_4_N/KB_0.3_, C_4_N/KB_0.45_, and C_4_N/rGO_0.45_, with 14 orders of magnitude increase for rGO-based composites. The specific surface areas of C_4_N, C_4_N/KB_0.3_, C_4_N/KB_0.45_, and C_4_N/rGO_0.45_ materials (Fig. 2h, j) were 587.8, 712.9, 798.5, and 388.6 m^2^ g^−1^, respectively, and the large specific surface areas will be beneficial for the infiltration of the electrolytes and the reaction kinetics. The pore sizes of C_4_N (Fig. 2i, k) were mainly distributed at 0.5 and 1.27 nm, basically consistent with the molecular structure shown in Fig. 1.

### Electrochemical Reaction Kinetics of Anode Materials

**Fig. 3:**
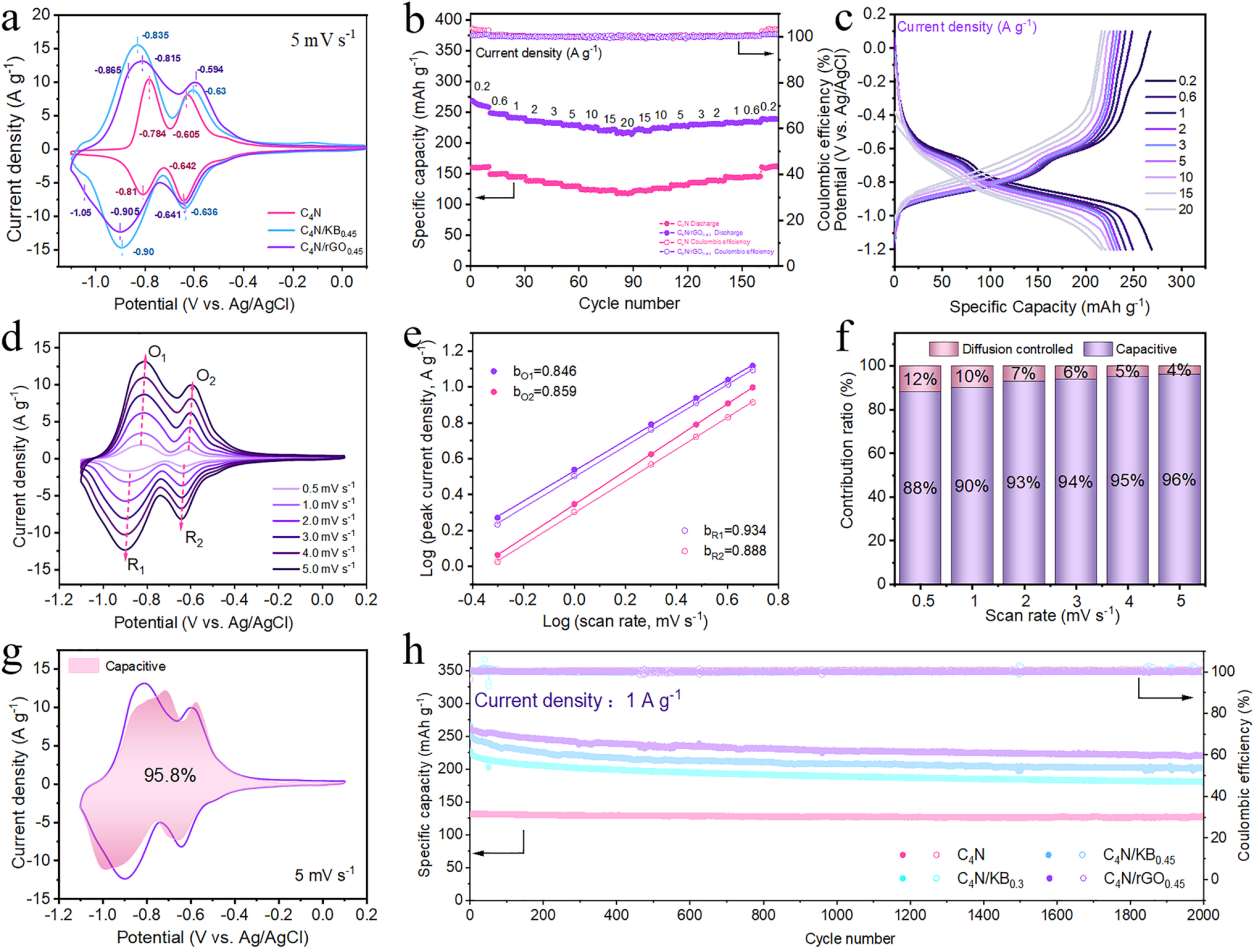
**a** CV curves at 5 mV s^−1^ and redox peak values for different anode materials. **b** Rate performance of the C_4_N and C_4_N/rGO in 2 M NaOH. Electrochemical performance and reaction kinetics of the C_4_N/rGO electrode in a three-electrode system. **c** Charge/discharge plots at various current densities. **d** CV profiles at different scan rates. **e** Linear logarithm relationship between the peak current densities and scan rates. **f** Contribution of the capacitor type to the total capacity. **g** Schematic diagram of capacitive contribution at 5 mV s^−1^. **h** Cycle stability at 1 A g^−1^ for different anode materials

Electrochemical properties of C_4_N were tested in various aqueous alkaline electrolytes (2 M LiOH, 2 M KOH, 2 M NaOH, 6 M NaOH and 10 M NaOH) (Fig. S6), and finally 2 M NaOH was chosen as the electrolyte owing to the moderate ionic size of Na^+^, the high elemental richness, low corrosion and its good compatibility with C_4_N electrode. A three-electrode system was used to study the electrochemical performances of C_4_N, C_4_N/KB_0.3_, C_4_N/KB_0.45_ and C_4_N/rGO_0.45_ electrode materials. The counter electrode used in the three-electrode test is activated carbon. The BET and SEM image are shown in the Figs. S7 and S8, where the specific surface area of activated carbon is 1722.7 m^2^ g^−1^. The cyclic voltammetry (CV) curves were analyzed in 2 M NaOH electrolyte using the C_4_N-based materials, activated carbon and Ag/AgCl as the working, counter and reference electrodes, respectively. Figure 3a showed the CV curves of different C_4_N-based electrodes in the potential range from 0.1 to − 1.1 V at a scan rate of 5 mV s^−1^, it can be seen that the redox peak current increased significantly after in situ compositing with conductive carbon materials and the redox potential decreased due to the enhanced active sites utilization. The lower reduction peak potentials are −0.81, −0.9 and −0.905 V for C_4_N, C_4_N/KB_0.45_, and C_4_N/rGO_0.45_, respectively. The rate performance tests show C_4_N/rGO_0.45_ has better Coulombic efficiency than KB-based composite electrode at large current densities (Figs. 3b and S9). The electrode sheets before and after cycling of C_4_N/rGO_0.45_ and C_4_N/KB_0.45_ are shown in Fig. S10. It can be seen that the C_4_N/KB_0.45_ has poorer film formation ability and the electrode sheet is more prone to cracking, but the morphology of C_4_N/rGO_0.45_ can be well maintained before and after cycling and the electrode presents better cycling stability (Fig. 3h). In addition to SEM, FTIR (Fig. S11) and TEM (Fig. S12) results of C_4_N/rGO_0.45_ electrode sheets before and after cycling also show that the material has excellent structural stability. Therefore, C_4_N/rGO_0.45_ is selected as the anode material of aqueous alkaline batteries for the follow-up study. In all the following descriptions, C_4_N/rGO is used instead of C_4_N/rGO_0.45_ for the sake of simplicity. The rate performance and galvanostatic charge/discharge (GCD) test displayed that C_4_N/rGO electrode owned significantly better rate performance than C_4_N alone (Fig. 3b, c). The specific capacities of C_4_N/rGO were 268.8, 248.9, 241.5, 235.7, 232.5, 229.1, 225.3, 220.4, and 216 mAh g^−1^ at current densities of 0.2, 0.6, 1, 2, 3, 5, 10, 15, and 20 A g^−1^, respectively. The capacity retention rate is 80.4% at 20 A g^−1^ with respect to the capacity at 0.2 A g^−1^. In contrast to the C_4_N electrode material (Fig. 3b), C_4_N/rGO delivers a specific capacity 167.9% more than the C_4_N electrode at 0.2 A g^−1^. CV curves of KB and rGO alone were also measured and did not show obvious redox peaks (Fig. S9), and their specific capacities are 9 and 12 mAh g^−1^, respectively, which is neglectable compared to those of C_4_N-based composites. The interfacial reaction dynamics between the electrode and the electrolyte was analyzed by EIS, and the Nyquist plots showed that the C_4_N/rGO had a smaller ohmic resistance and charge-transfer resistance than C_4_N, meaning a better electrode conductivity and faster reaction kinetics (Fig. S13).

To further explore the charge storage kinetics, the electrode C_4_N/rGO was subjected to a continuous CV test with the scan rate increasing from 0.5 to 5 mV s^−1^ in 2 M NaOH electrolyte, and the larger scan rate corresponded to the larger peak area of CV curve (Fig. 3d). In general, the relationship between the peak current (*i*) and the scan rate (*v*) is described according to the following equation [40]:

1 $$ {\text{i}}\;{ = }\;{\text{av}}^{{\text{b}}} $$

Through the plot of log (*i*) versus log (*ν*) (Fig. 3e), the b values of the four labelled redox peaks in Fig. 3d were calculated to be 0.846, 0.859, 0.934, and 0.888, respectively, indicating that the redox process of C_4_N/rGO was controlled by the capacitive behavior and ionic diffusion together. As the scan rate increased from 0.5 to 5 mV s^−1^, the percentage of capacitive contribution increased from 88% to 96% (Fig. 3f, g). It indicated that the charge storage of C_4_N/rGO is mainly dominated by the pseudocapacitive effect at the interface, which ensured the fast charge transfer kinetics and high-rate performance. In addition, we have analysed the diffusion coefficients of C_4_N/rGO materials by the Galvanostatic Intermittent Titration Technique (GITT), a pulse-constant-current-relaxation cyclic process, where pulse refers to a short current passage and relaxation refers to the absence of current passage. From the GITT curves and diffusion coefficient plots (Fig. S14), it could be seen that the diffusion coefficients of the material were in the range of 10^–10^–10^–8^, which once again proved that the material has a very good ion transport capacity and fast charge transfer kinetics. After 2,000 cycles at 1 A g^−1^, C_4_N/rGO_0.45_ still remained a high specific capacity of 220.5 mAh g^−1^ and the capacity retention was 84.2%, while those of C_4_N/KB_0.45_ were 202.2 mAh g^−1^ and 76%, respectively, confirming the more excellent cycling stability of C_4_N/rGO in alkaline aqueous electrolyte than C_4_N/KB (Fig. 3h).

### Electrochemical Charge Storage Mechanism of Anode Materials

**Fig. 4:**
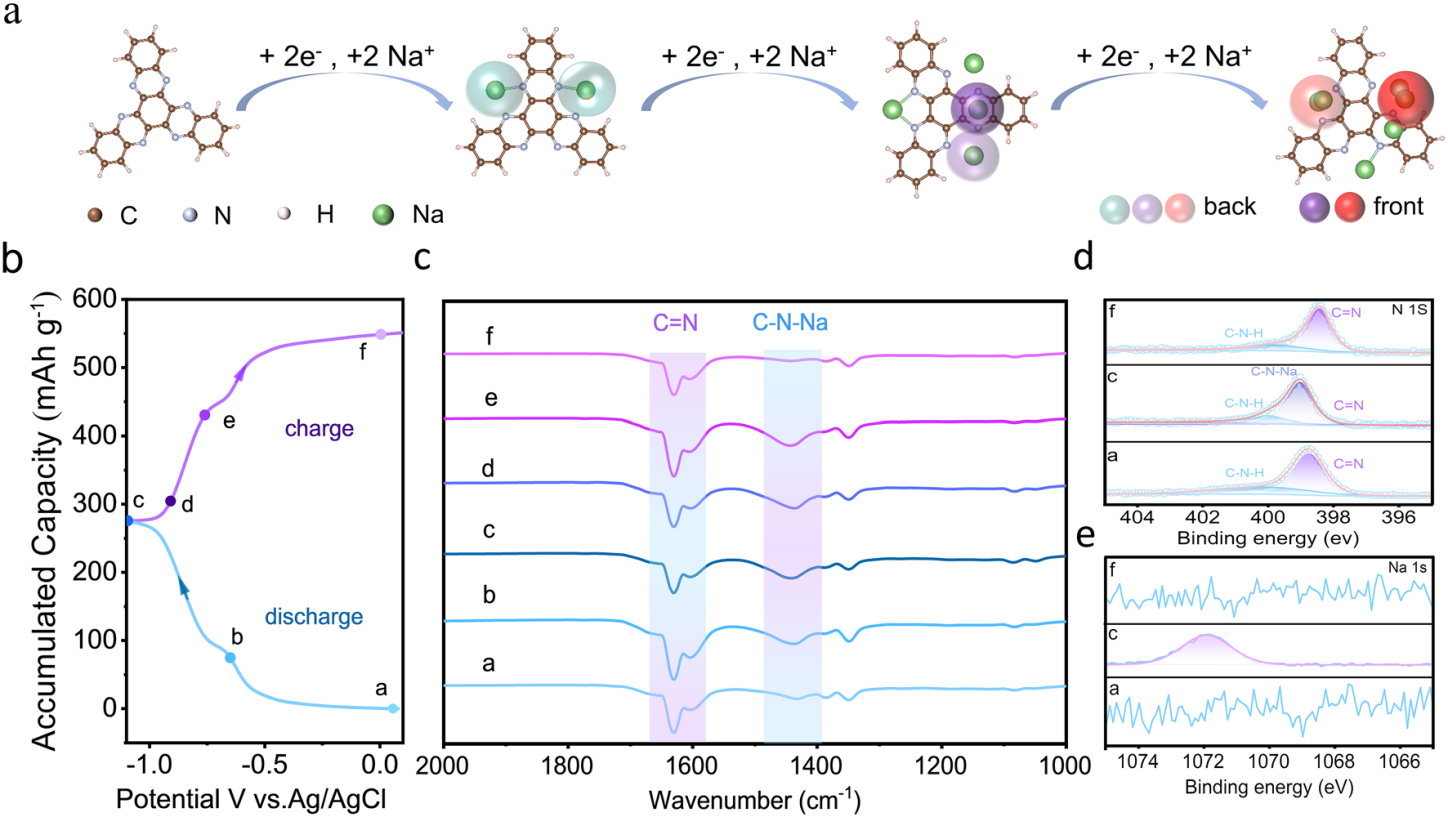
**a** Sodification pathway of the C_4_N obtained from simulations. Structural evolution during charge/ discharge. **b** Galvanostatic charge/ discharge profile at a rate of 1 A g^−1^ and the selected points for the ex-situ tests. **c** Ex-situ FT-IR spectra under different potential states. **d** XPS spectra of N 1*s* at different potentials. **e** XPS spectra of Na 1*s* at different potentials

To speculate the active sites for electrophilic and nucleophilic reactions, molecular electrostatic potential (MESP) of the smallest repeating unit of C_4_N is calculated and shown in Fig. S15. The pyrazine nitrogen atoms in the blue region with more negative MESPs have higher electronegativities, and are more prone to attracting the cation Na^+^. To further understand charge storage mechanism of C_4_N, we apply DFT [22] to simulate the sodiation process of C_4_N. The optimal structures of C_4_N–xNa (x = 2, 4, 6) and their Gibbs free energy changes (ΔG) are shown in Fig. S16. Various structural changes during the sodiation of C_4_N are schematically shown in Fig. 4a, and the side view of the structures are shown in Fig. S17. During the ion insertion process, the structures evolve from C_4_N to C_4_N–2Na, C_4_N–4Na, and C_4_N–6Na, respectively (x = 2, 4, 6) in a highly reversible manner, and ΔG values for each step are negative, which implies that six nitrogen active sites can be fully sodiated to deliver a specific capacity of 422 mAh g^−1^ in theory.

To further verify how the C_4_N anode material stores and releases the charge, we performed a series of ex-situ characterizations. Figure 4b displayed the charge/discharge curves of C_4_N/rGO, and the chemical structures of C_4_N/rGO electrodes at different charge/discharge states were checked by the ex-situ FT-IR and XPS. The FTIR spectra of the electrodes at different potentials were shown in Fig. 4c, and the intensity of the peak around 1634 cm^−1^ assigned to C=N bond at the initial discharge stage (point a) was higher than that at other stages. During the discharge process, the potential gradually decreased and the peak intensity of the C=N bond gradually weakened, indicating that the C=N bond was reduced, and a new peak at 1440 cm^−1^ corresponding to the C–N–Na bond in the FT-IR spectrum appeared and gradually strengthened, indicating that sodium ion storage depends on the conversion of C=N bond to C–N–Na bond. Contrary to the discharge process, the peak strength of the C=N bond gradually increased during the charging process along with the weakening of the C–N–Na bond, and when the charging was completed, the strength of the C=N bond was maximized at the point f, and the peak of the C–N–Na bond almost disappeared. Figure 4d showed the high-resolution XPS spectra of N 1*s*. The fitted subpeak at 398.8 eV assigned to C=N decreased and the subpeak assigned to C–N–Na increased during the discharge process, and the change trend was reversed in the charging process [41]. Figure 4e showed the XPS spectrum of Na 1*s*, and at point c where the discharge ends, the signal peak of sodium was the strongest, confirming the insertion of sodium ions. We have also carried out ex-situ characterizations of C_4_N/rGO electrodes undergoing charging and discharging by taking SEM and EDS mapping images (Fig. S18) of the electrodes at the original state (a), discharged to −1.1 V (c) and charged to 0 V (f), respectively. We could see that when charging to point c, the Na content increased from 0 to 12.01 wt%, and when discharging to point f, the sodium content decreased again to 0.83 wt%. This result was consistent with FTIR and XPS results. All these results well match with the calculated results, and verifies the high reversibility of Na^+^ storage in the C_4_N-based electrode.

### Electrochemical Behavior of the Full Battery

**Fig. 5:**
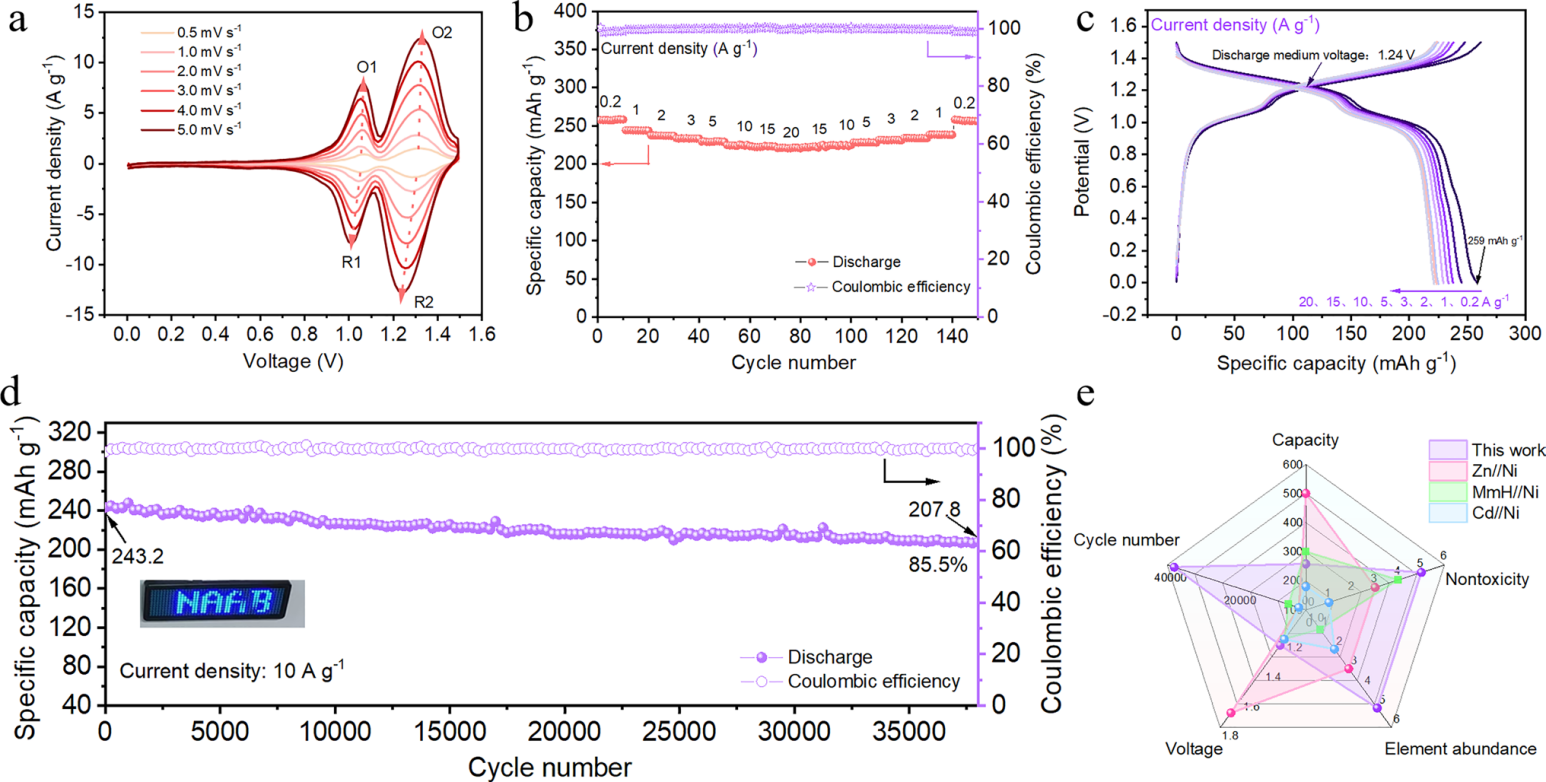
Electrochemical characterizations of the full cells of C_4_N/rGO//Ni(OH)_2_. **a** CV profiles of the full cell at 0.5–5 mV s^−1^ in 2 M NaOH. **b** Rate performance of the full cell. **c** GCD curves at different current densities. **d** Cycle stability at 10 A g^−1^ in 2 M NaOH. **e** Comprehensive performance evaluation, including the capacity, cycle number, voltage, nontoxicity and element abundance between the conventional Ni-based batteries (Zn//Ni, MmH//Ni and Cd//Ni) [11–15] and C_4_N/rGO//Ni(OH)_2_. (The capacity is calculated according to the anode material; nontoxicity and element abundance indicators are graded from 1 to 5)

The full battery was assembled with C_4_N/rGO material as the anode, β-Ni(OH)_2_ (Fig. S19) as the cathode, and 2 M NaOH as the electrolyte. The CV diagram of the cell (Fig. 5a) showed two pairs of distinct oxidation and reduction peaks at different sweep rates, and the electrochemical reaction kinetics of the full cell were investigated and analyzed by the Eq. 1 and the following formula:

2 $$ {\text{i}} = {\text{k}}_{1} {\text{v}} + {\text{k}}_{2} {\text{v}}^{1/2} $$

where k_1_v and k_2_v^1/2^ stand for capacitive and diffusion-controlled capacity contribution, respectively (Fig.S20). The results showed that the electrochemical behavior of the full battery was mainly dominated by the pseudo capacitance effect at the interface when the scan rate was 5 mV s^−1^ (Fig. S20c), and the capacitive contribution accounted for 93.1% of the total capacity. The GCD curves and rate performance of the full cell are shown in Fig. 5b, c. The specific capacities based on the mass of the negative electrode were 259, 245.1, 237.6, 233.9, 229.4, 225, 223.3, and 221.3 mAh g^−1^ at current densities of 0.2, 1, 2, 3, 5, 10, 15, and 20 A g^−1^. The discharge medium voltage of the full cell was 1.24 V at 0.2 A g^−1^ and the energy density was 134 Wh Kg^−1^ based on the total mass of the positive and negative electrodes. Figure 5d showed the long-term cycling performance of the C_4_N/rGO//Ni(OH)_2_ full cell at 10 A g^−1^, and the capacity decayed from 243.2 to 207.8 mAh g^−1^ after cycling for 38,000 cycles, with a capacity retention rate of 85.5% and the average capacity decay rate of about 0.00038% throughout the cycling process, demonstrating super cycling stability. A comparison of this battery with conventional commercial alkaline nickel-based batteries is shown in Table S3. This work avoids the use of toxic cadmium metal and expensive hydrogen storage alloys, and has comprehensive performance advantages in terms of toxicity, cycling stability, energy density, and element abundance (Fig. 5e).

### Alkali-Resistant Antifreeze Additives and Full Cell for Wide Temperature Range Applications

In order to meet the requirements of large-scale energy storage and wide temperature range application, the aqueous alkaline electrolyte in this work was adjusted to enable stable operation of C_4_N/rGO//Ni(OH)_2_ under ultra-low temperature conditions. Among the antifreeze additives, dimethyl sulfoxide (DMSO) is a low-cost high-polarity aprotic organic solvent with good stability under alkaline conditions. It has the strong ability to form hydrogen bonds with water molecules and thus can limit the activity of water molecules [42]. However, it is undeniable that the addition of DMSO will increase the combustibility of electrolytes and thus we use a low concentration of DMSO to minimize the negative impact of antifreeze additive on the aqueous electrolyte.

**Fig. 6:**
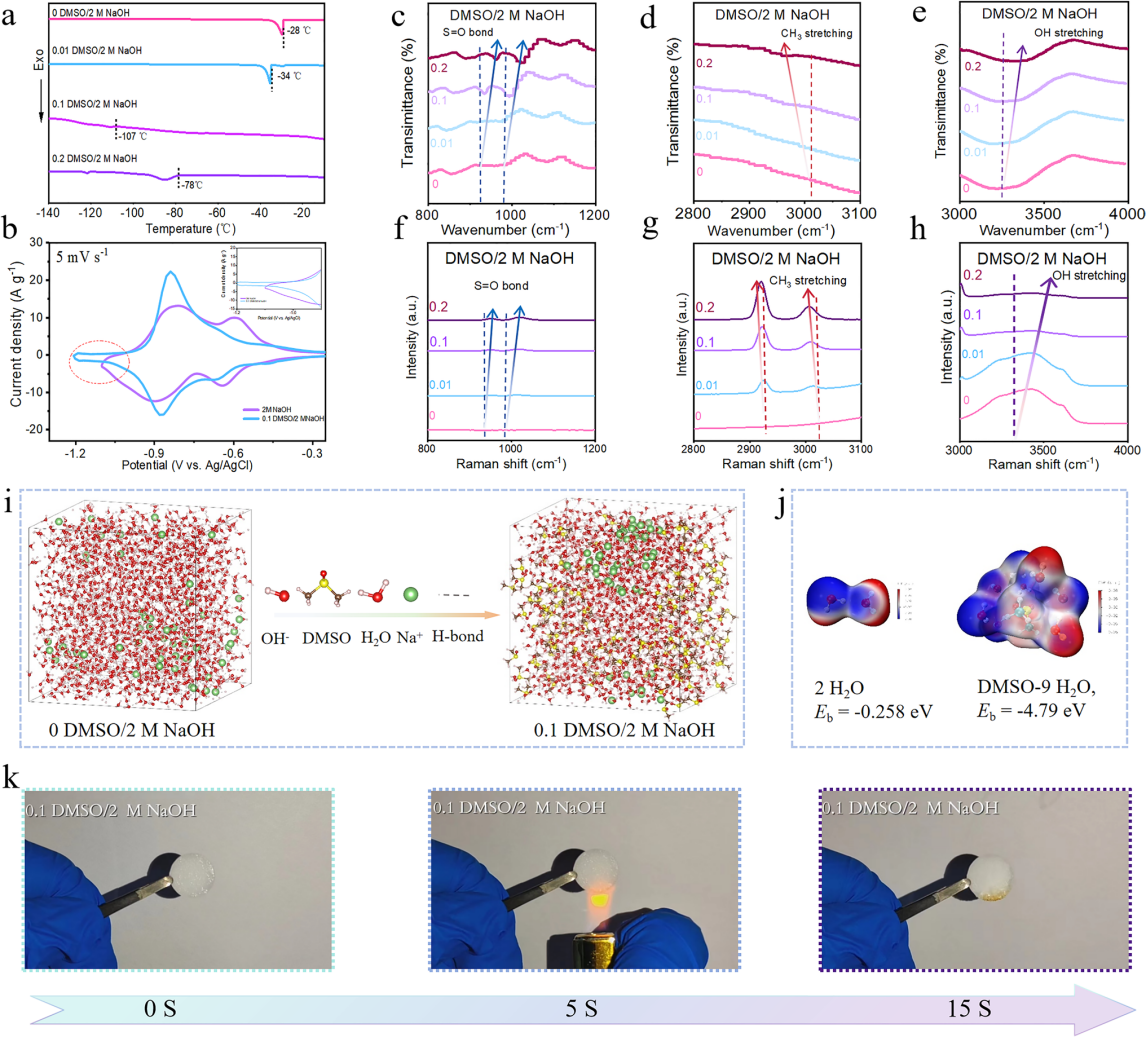
Low-temperature properties of electrolytes with different molar fractions of DMSO in 2 M NaOH. **a** DSC test from  −140 to −10 °C at a heating rate of 5 °C min^−1^. **b** CV curves at 5 mV s^−1^ of C_4_N/rGO electrodes tested in different electrolytes. FTIR spectra for **c** S=O bond of DMSO, **d** CH_3_ stretching modes of DMSO and **e** OH stretching vibration of water. Raman spectra for **f** S=O bond of DMSO, **g** CH_3_ stretching modes of DMSO and **h** OH stretching vibration of water. **i** Snapshot of MD simulations of electrolytes with molar fraction of DMSO at 0 and 0.1. **j** Ionic interactions of electrolytes with and without 0.1 DMSO. **k** Combustion process of 0.1 DMSO/2 M NaOH electrolyte

We modulated the 2 M NaOH electrolyte with different molar fractions of DMSO (0, 0.01, 0.1, 0.2) and investigated their properties by both experiments and theoretical calculations. Differential scanning calorimetry (DSC) results in Fig. 6a showed that the freezing point of the electrolyte first decreased and then increased as the molar fraction of dimethyl sulfoxide increased. When the molar fraction of DMSO added in 2 M NaOH was 0.1, the freezing point was the lowest at −107 °C. Figure S21 showed the optical photographs of the different electrolytes before and after being placed at −70 °C for 0, 2, and 12 h. Only the 0.1 DMSO/2 M NaOH electrolyte maintained its fluidity. In addition, we found that when the electrode materials C_4_N/rGO were subjected to CV tests under 2 M NaOH and 0.1 DMSO/2 M NaOH electrolytes, respectively, the hydrogen evolution phenomenon of the latter was significantly weakened [43] and the electrochemical stability window was widened (Fig. 6b). We performed FTIR and Raman spectroscopy on the different electrolytes (Fig. S22). As the molar fraction of DMSO increased, the peak of the S=O bond was gradually blue-shifted (Fig. 6c) [10, 42], the CH_3_ stretching was gradually red-shifted (Fig. 6d) [29, 43], and the peak of the OH stretching vibration was gradually blue-shifted (Fig. 6e), indicating that the hydrogen bonding interactions between water molecules were weakened due to the introduction of DMSO. Furthermore, the Raman spectroscopy (Fig. 6f–h) results were in agreement with the FTIR results, further confirming DMSO could establish strong hydrogen bond interaction with water molecules to break the hydrogen bond networks of water molecules. The weaken hydrogen bond interaction between water molecules would inhibit the nucleation and crystallization of ice by increasing the translational energy gap of water molecules, thus serving to lower the freezing point.

A snapshot of the MD simulation is shown in Fig. 6i. The calculations show that the number of H-bonds decreases considerably with the introduction of DMSO at a molar fraction of 0.1 (Table S4). As shown in Fig. 6j, the calculations indicate that the binding energy between DMSO and H_2_O is stronger than that between water molecules, which again suggests that DMSO is capable of disrupting the hydrogen bonding interaction of water and inhibits the freezing of water. However, a significant increase in the molar fraction of DMSO will lead to a rise in the Ewald energy, which will increase the friction between the aggregation units of DMSO/2 M NaOH electrolytes. In consequence, this may induce an increase in the freezing point of the electrolyte, which is not beneficial for low-temperature operation of the electrolyte [29]. It is therefore crucial to choose the right molar fraction to ensure suitable interactions between the DMSO and water molecules so that the system can function smoothly at the expected temperature. In addition, the combustion test indicated that 0.1 DMSO/2 M NaOH electrolyte is non-inflammable (Figs. 6k and S23), confirming the high safety of such aqueous alkaline battery while maintaining low freezing point and wide operation voltage window.

The electrode material C_4_N/rGO was subjected to a continuous CV test in 0.1 DMSO/2 M NaOH electrolyte, and the scan rate was increased from 0.5 to 5 mV s^−1^. According to Eq. 1, the b values were calculated and used to determine capacitive contribution (Fig. S24). The results showed that the electrochemical behavior of the electrode material in this electrolyte was similar to that in 2 M NaOH electrolyte, and the redox process is controlled by both capacitive behavior and ion diffusion. Nyquist plots (Fig. S24e) showed that C_4_N/rGO also had a small charge transfer resistance. The GCD curves (Fig. S24f), rate performance (Fig. S24g), and long-cycle performance (Fig.S24h) indicated that the electrochemical performance of the anode material at room temperature was almost unaffected by the addition of DMSO, and the addition of DMSO helped to inhibit the hydrogen precipitation at the anode (Fig. S25). In addition, it can be seen from Fig. S26 hat the electrolyte with 0.1 DMSO/2 M NaOH has a better wettability with the electrode sheet, which explains the slight difference between the CV and GCD plots. When the current density was 0.2 A g^−1^, the specific capacity of the anode material was 274.7 mAh g^−1^, which was higher than that in the electrolyte without DMSO, and the electrode also displayed an excellent cycling stability.

**Fig. 7:**
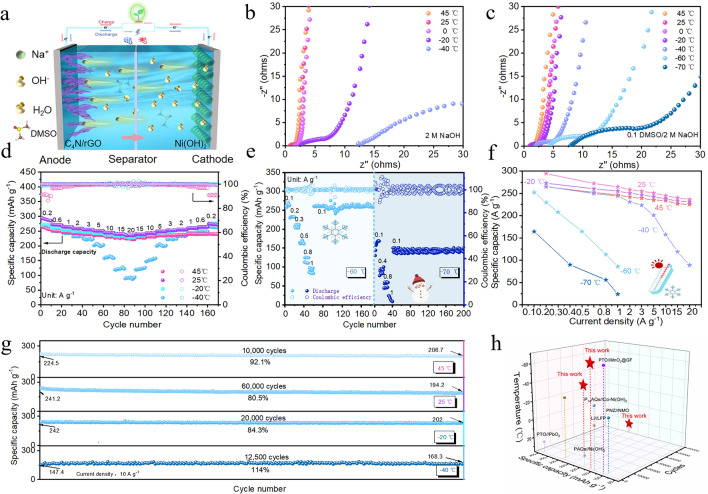
Electrochemical characterizations of the full cell of C_4_N/rGO//Ni(OH)_2_ in a wide temperature range. **a** Schematic diagram of the full battery in the electrolyte of 0.1 DMSO/2 M NaOH. Nyquist impedance plots of full cells with different electrolytes at different temperatures: **b** 2 M NaOH and **c** 0.1 DMSO/2 M NaOH. **d** Rate performance of the full cell at different temperatures (−40, −20, 25, 45 °C). **e** Rate performance and cycle stability of the full cell at  −60 and −70 °C. **f** Specific capacities of full cells at different temperatures and different current densities. **g** Long cycle performance of the full battery at different temperatures (−40, −20, 25, 45 °C). **h** Comparison of electrochemical properties of different batteries (acid, neutral and alkaline)

Figure 7a showed the schematic diagram of a C_4_N/rGO//Ni(OH)_2_ full cell with C_4_N/rGO as the anode, Ni(OH)_2_ as the cathode, and 0.1 DMSO/2 M NaOH as the electrolyte. Continuous CV tests were performed on the full cell at room temperature, and the CV curves were analyzed (Fig. S27). The results showed that the maximum stable voltage window of the electrolyte was elevated from the original 1.5 to 1.6 V due to the fact that water molecules in the original solvation shell of the sodium ions was partially replaced by DMSO, resulting in the less contact of water molecules with the electrode surface during the charging and discharging process after the addition of DMSO, which inhibited the hydrogen precipitation reaction [43]. The extended electrochemical window will help to further increase the capacity of the full cell. The impedances of full cells with electrolytes of 2 M NaOH and 0.1 DMSO/2 M NaOH at different temperatures (45, 25, 0, −20, −40, −60, and −70 °C) (Figs. 7b, c and S28) were tested, and all Nyquist plots showed the characteristic semicircles in the high-frequency region and nearly vertical lines in the low-frequency region. From the graphs, we could see that the impedance of the full cell with the electrolyte without DMSO added was smaller than that with DMSO added above 0 °C. It could be seen that the electrolyte resistance increased as the temperature decreased. This was because the viscosity of the electrolyte gradually increased as the temperature decreased, which results in the decrease of the ion migration rate. However, the rate of decrease in the conductivity of the 0.1 DMSO/2 M NaOH electrolyte at ultra-low temperatures (below −20 °C) was less than that in 2 M NaOH electrolyte, because the electrolyte had a lower freezing point, smaller viscosity, and thus higher ionic conductivity at low temperatures. The test results demonstrated that the electrolyte had an ionic conductivity of 0.014 S cm^−1^ at −70 °C, allowing the full cell to operate at ultra-low temperatures.

The electrochemical performances of the full cells were further examined by GCD tests in both electrolyte systems. At −20 °C, the C_4_N/rGO//Ni(OH)_2_ cell with 0.1 DMSO/2 M NaOH as the electrolyte showed superior rate performance (Fig. S29), high specific capacity and stable Coulombic efficiency. At 0.2 A g^−1^, the specific capacity of the cell was 273.1 mAh g^−1^ and the Coulombic efficiency gets close to100%. Even at a high current density of 20 A g^−1^, it still had a specific capacity of 231.6 mAh g^−1^. However, the specific capacity of the cell with 2 M NaOH electrolyte was only 192.1 mAh g^−1^ at 1 A g^−1^, much lower than 255.5 mAh g^−1^ for the cell with 0.1 DMSO/2 M NaOH electrolyte, and the Coulombic efficiency was only about 93%. At room temperature, the specific capacities of the full cell with 0.1 DMSO/2 M NaOH electrolyte were 294.6, 272.4, 264.9, 258.7, 254.9, 251, 244.8, 240.2, and 236.6 mAh g ^−1^ at room temperature at 0.2, 1, 2, 3, 5, 10, 15, and 20 A g^−1^, and the specific capacity at 20 A g^−1^ t is 80.3% of that at 0.2 A g^−1^, indicating an excellent rate performance. At -40 °C, the cell can deliver a specific capacity of 265.7 mAh g^−1^ at 0.2 A g^−1^ with an approximate 100% Coulombic efficiency and can perform normal charge/discharge operation at 20 A g^−1^ (Fig. 7d). Even at −60 °C, a high specific capacity of 252.3 mAh g^−1^ at 0.1 A g^−1^ was still achieved with an 85.6% capacity retention rate relative to the capacity at room temperature. When the temperature dropped to −70 °C, a capacity of 164.6 mAh g^−1^ at 0.1 A g^−1^ was still maintained. All the above results indicate that the cell has excellent low temperature performance (Fig. 7e). The capacities of the full cell at various temperatures and current densities are shown in Fig. 7f. The long cycling test (Fig. 7g) results demonstrated that the initial specific capacity of full cell is 224.5 mAh g^−1^ at 45 °C, and the capacity retention rate was 92.1% after 10,000 cycles. When the temperatures are 25, −20, and −40 °C, the capacity retention rates are 80.5% after 60,000 cycles, 84.3% after 20,000 cycles and 114% after 12,500 cycles, respectively.

Above all, the results show that the C_4_N/rGO//Ni(OH)_2_ full cell has ultra-high cycling stability over a wide temperature range from −70 to 45 °C. Thus, the use of our modulated electrolyte brings about the possibility for the development of C_4_N/rGO//Ni(OH)_2_ cells that operate at extremely low temperatures. Table S5 summarizes the electrochemical performances of the previously reported aqueous batteries under different electrolyte and electrode systems, and it can be seen that the cycling stability and low-temperature performance of the battery in the present work are at a high level. Due to the high ionic conductivity of the modulated alkali-tolerant low-temperature electrolyte at ultra-low temperature and the high capacity and superb stability of the C_4_N/rGO anode, the room-and low-temperature performance (194.2 mAh g^−1^ after 60,000 cycles@10 A g^−1^ at 25 °C, 202 mAh g^−1^ after 12,500 cycles@10 A g^−1^ at −40 °C and 150 mAh g^−1^ after 160 cycles@0.1 A g^−1^ at −70 °C) of full cell are superior to most reported batteries (Fig. 7h), including the acidic PTO//PbO_2_ [15] and PTO//MnO_2_@GF [44], neutral Li//LFP [45], and Zn//V_2_O_5_ [46] as well as alkaline PAQs//Ni(OH)_2_ [15], P_14_AQs//Co–Ni(OH)_2_ [47], and PNZ//NMO [48]. This work is expected to advance the development of high-performance organic anode materials to enable aqueous alkaline batteries with high energy density, long service life, and wide operation temperature range.

## Conclusions

In summary, this paper reports the in-situ growth of porous C_4_N conjugated polymer material on conductive carbon substrates and the applications as the anode materials of aqueous alkaline sodium-ion batteries. The effect of the carbon substrates on the electrochemical performance of C_4_N in aqueous alkaline sodium-ion batteries was investigated. Due to high integration, large porosity and good conductivity, C_4_N/rGO can directly be prepared into the electrode only with PVDF adhesives, and shows a low redox potential (−0.905 V vs. Ag/AgCl), high sodium ion storage capacity (268.8 mAh g^−1^ at 0.2 A g^−1^), good stability (84.2% after 2000 cycles at 1 A g^−1^) and outstanding rate capability (216 mAh g^−1^ at 20 A g^−1^) in 2 M NaOH electrolyte. The Na^+^ storage mechanism of the anode materials was verified using XPS, FT-IR, SEM, EDS and DFT calculations, and the highly reversible conversion between C=N and C–N–Na bonds is responsible for Na^+^ storage and release. The assembled C_4_N/rGO//Ni(OH)_2_ full cell has a high energy density (134 Wh Kg^−1^) and outstanding cycling stability (38,000 cycles at a current density of 10 A g^−1^), which provides a comprehensive performance advantage over the conventional commercial nickel-based batteries. Furthermore, an antifreeze aqueous alkaline electrolyte (0.1 DMSO/2 M NaOH) was developed by adding a small amount of alkali-resistant DMSO into 2 M NaOH. As a consequence, the C_4_N/rGO//Ni(OH)_2_ full cell delivers a high energy density (147.3 Wh Kg^−1^ at 25 °C) and ultra-stable cycling over a wide temperature range from −70 to 45 °C. At 45 °C, the battery can exhibit an initial specific capacity of 224.5 mAh g^−1^ and 92.1% capacity retention after 10,000 cycles at 10 A g^−1^. At −40 °C, a specific capacity of 168.3 mAh g^−1^ can be retained at 10 A g^−1^ after 12,500 cycles. Even at −70 °C, the battery can still deliver a specific capacity of about 164.6 mAh g^−1^ and a capacity retention rate of nearly 91% after 160 cycles at 0.1 A g^−1^. This work sheds light on the study of high-performance organic anode materials and antifreeze electrolytes for aqueous alkaline batteries and will pave the way for the development of wide-temperature-range aqueous alkaline batteries with both high energy density and good cycling stability. 

## Supplementary Information

Below is the link to the electronic supplementary material.

Supplementary file1 (DOCX 21408 KB)
