# Supplementary material for: An Ultra-Stable, High-Energy and Wide-Temperature-Range Aqueous Alkaline Sodium-Ion Battery with the Microporous C4N/rGO Anode
Source: Nanomicro Lett. 2025 Feb 24;17:158. doi: 10.1007/s40820-024-01589-w (PMC11850668; doi:10.1007/s40820-024-01589-w)
Supplement: Supplementary file 1 — Supplementary file1 (DOCX 21408 KB) [file 40820_2024_1589_MOESM1_ESM.docx]

Supporting Information for

**An Ultra-Stable, High-Energy and Wide-Temperature-Range Aqueous Alkaline Sodium-Ion Battery** **with the Microporous C_4_N/rGO Anode**

Mengxiao Li^1^, Rui Li^2,3^, Huige Ma^2, 3^, Mingsheng Yang^1^, Yujie Dai^2,3^, HaiPing Yu^2,3^, Yuxin Hao^2,3^, Zhihui Wang^1^, Bei Wang^2,3^, Mingjun Hu^1,^*, Jun Yang^2,3,4,^*

^1^ School of Materials Science and Engineering, Beihang University, Beijing100191, P. R. China

^2^ Beijing Institute of Nanoenergy & Nanosystems, Chinese Academy of Sciences, Beijing 101400, P. R. China

^3^ School of Nanoscience and Engineering, University of Chinese Academy of Sciences, Beijing 100049, P. R. China

^4^ ShenSi Lab, Shenzhen Institute for Advanced Study, University of Electronic Science and Technology of China, Shenzhen 518110, P. R. China

^*^Corresponding authors. E-mail: [mingjunhu@buaa.edu.cn](mailto:mingjunhu@buaa.edu.cn) (Mingjun Hu); [yangjun@binn.cas.cn](mailto:yangjun@binn.cas.cn) (Jun Yang)

**Supplementary Figures and Tables**


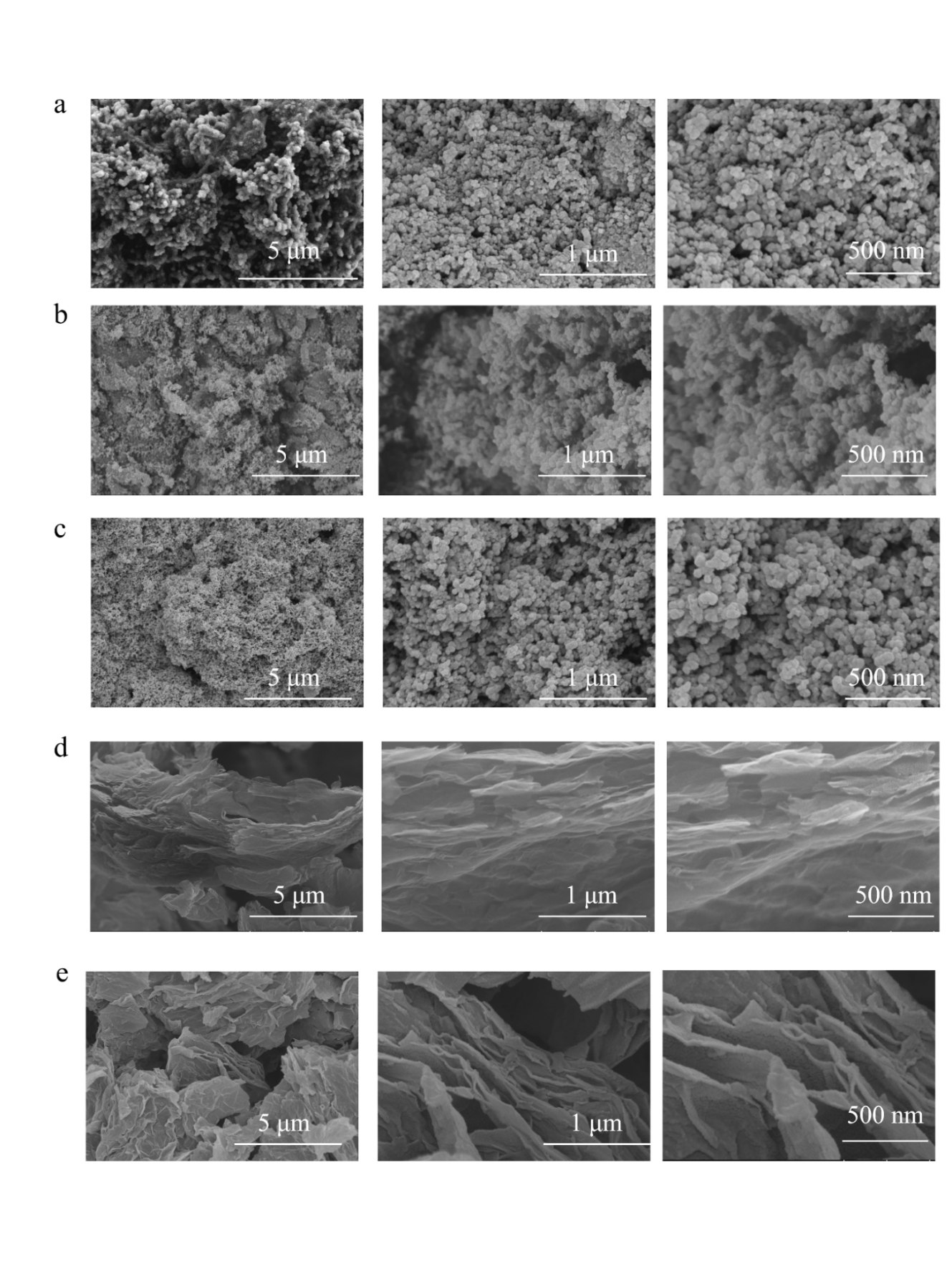


**Fig. S1** SEM images of (**a**) C_4_N, (**b**) KB, (**c**) C_4_N/KB_0.45_, (**d**) rGO and (**e**) C_4_N/rGO_0.45_


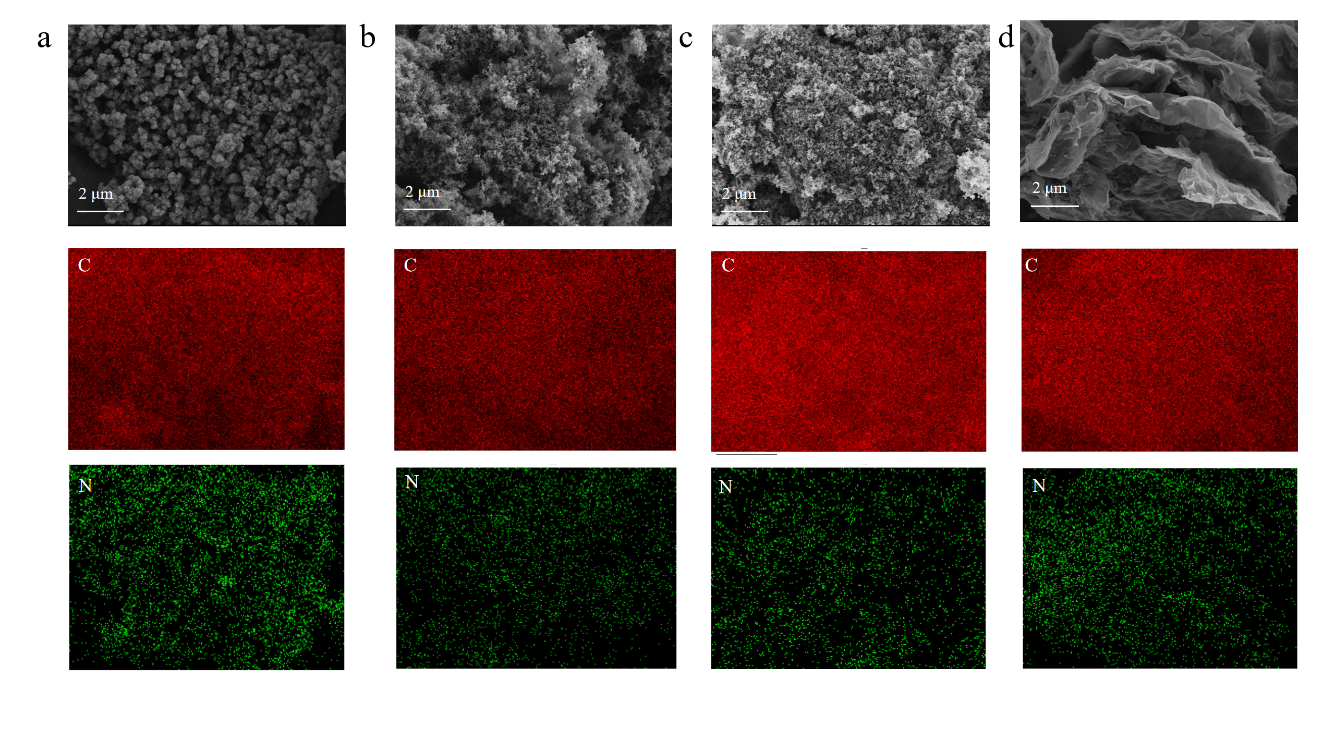


**Fig. S****2** SEM images and EDS mapping images of (**a**) C_4_N, (**b**) C_4_N/KB_0.3_, (**c**) C_4_N/KB_0.45_ and (**d**) C_4_N/rGO_0.45_


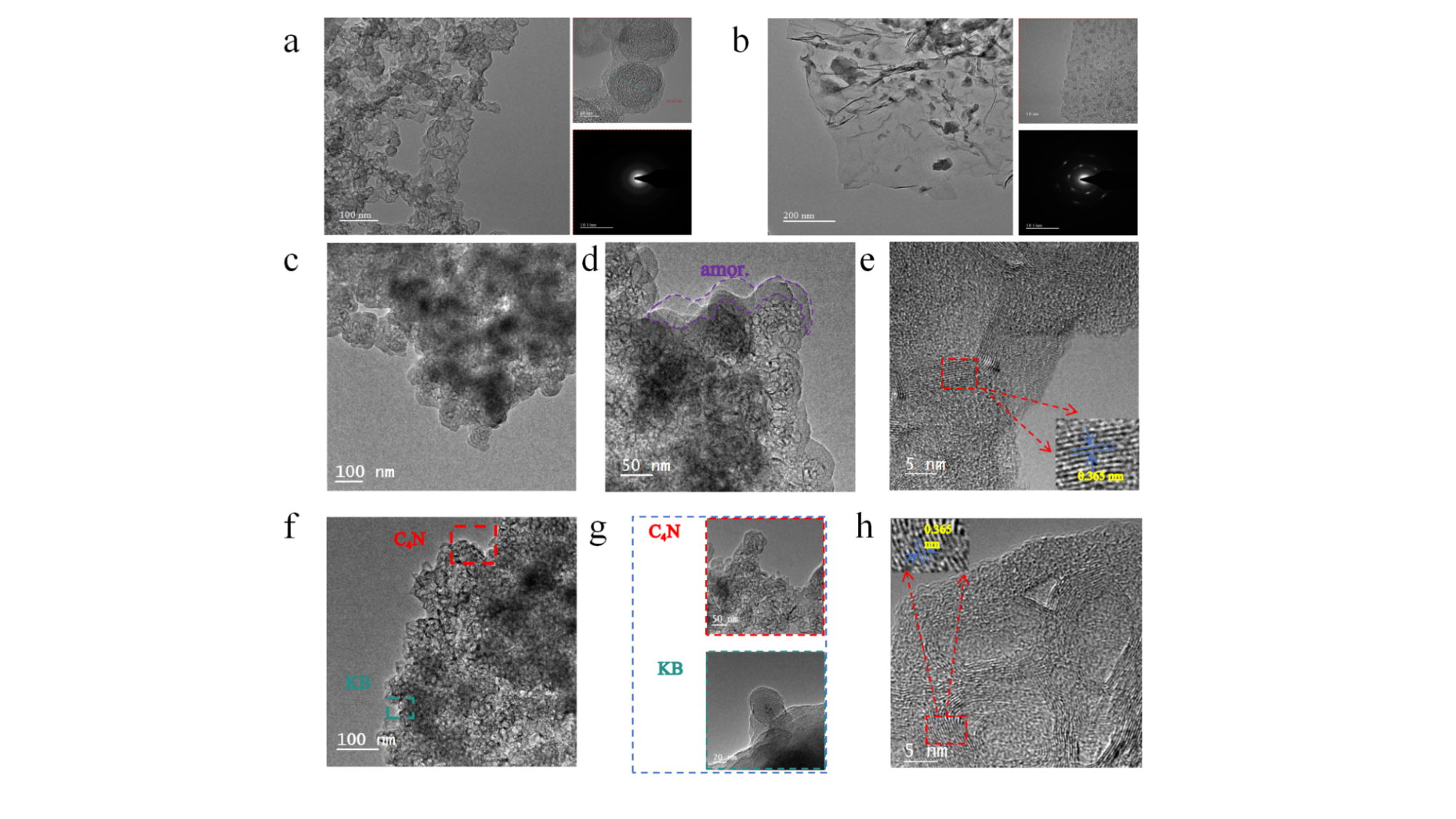


**Fig. S3** TEM images of (**a**) KB, (**b**) rGO and (**c**) C_4_N. (**d**) Magnified TEM image of the C_4_N. HRTEM image of (**e**) C_4_N. TEM images of (**f**) C_4_N/KB_0.3_. (**g**) Magnified TEM images of C_4_N and KB corresponding to the selected areas of f. HRTEM image of (**h**) C_4_N/KB_0.3_


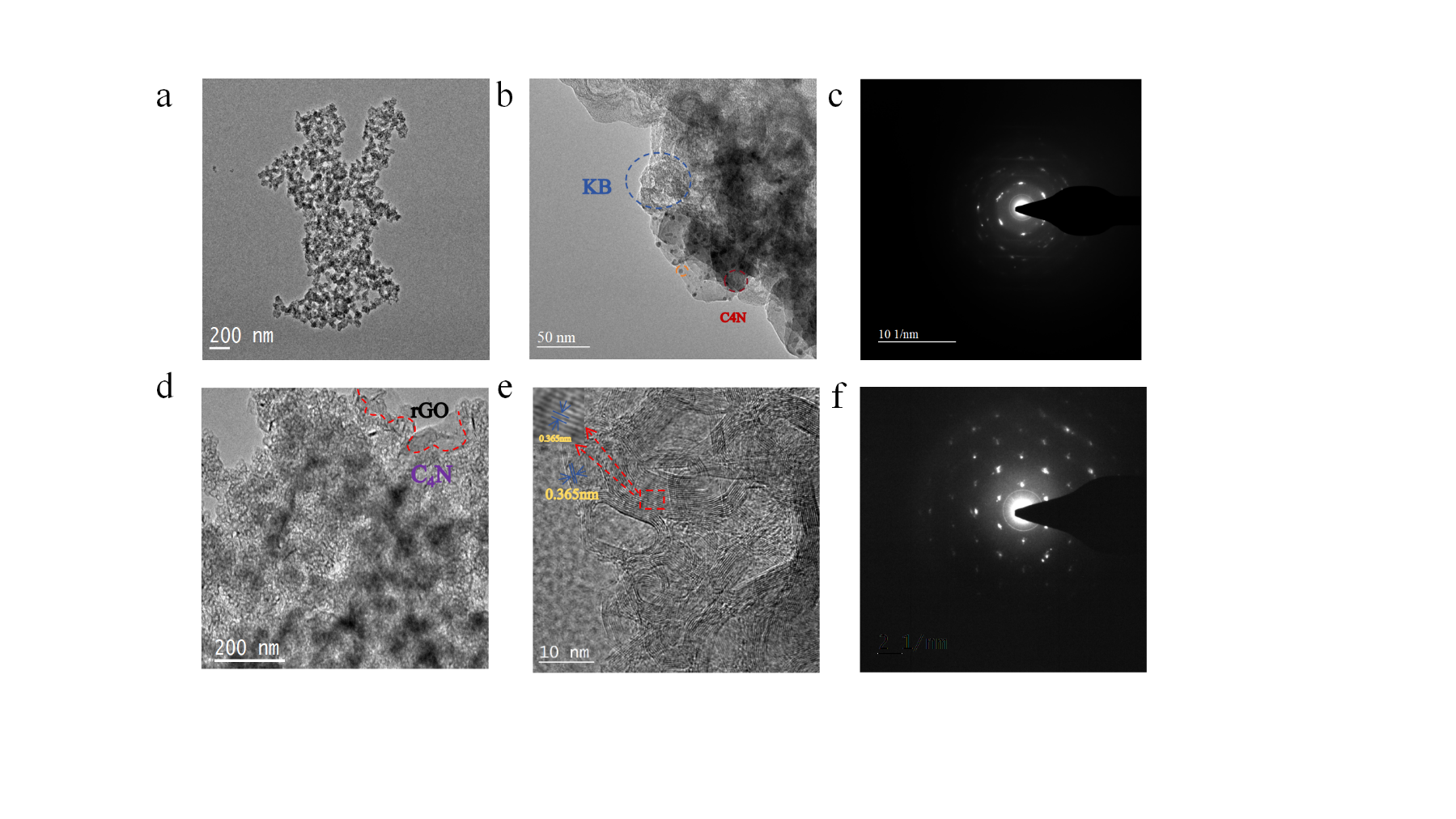


**Fig. S4** (**a**) TEM, (**b**) magnified TEM and (**c**) Electron diffraction pattern images of the C_4_N/KB_0.45_. (**d**) TEM, (**e**) HRTEM and (**f**) Electron diffraction pattern images of the C_4_N/rGO_0.45_


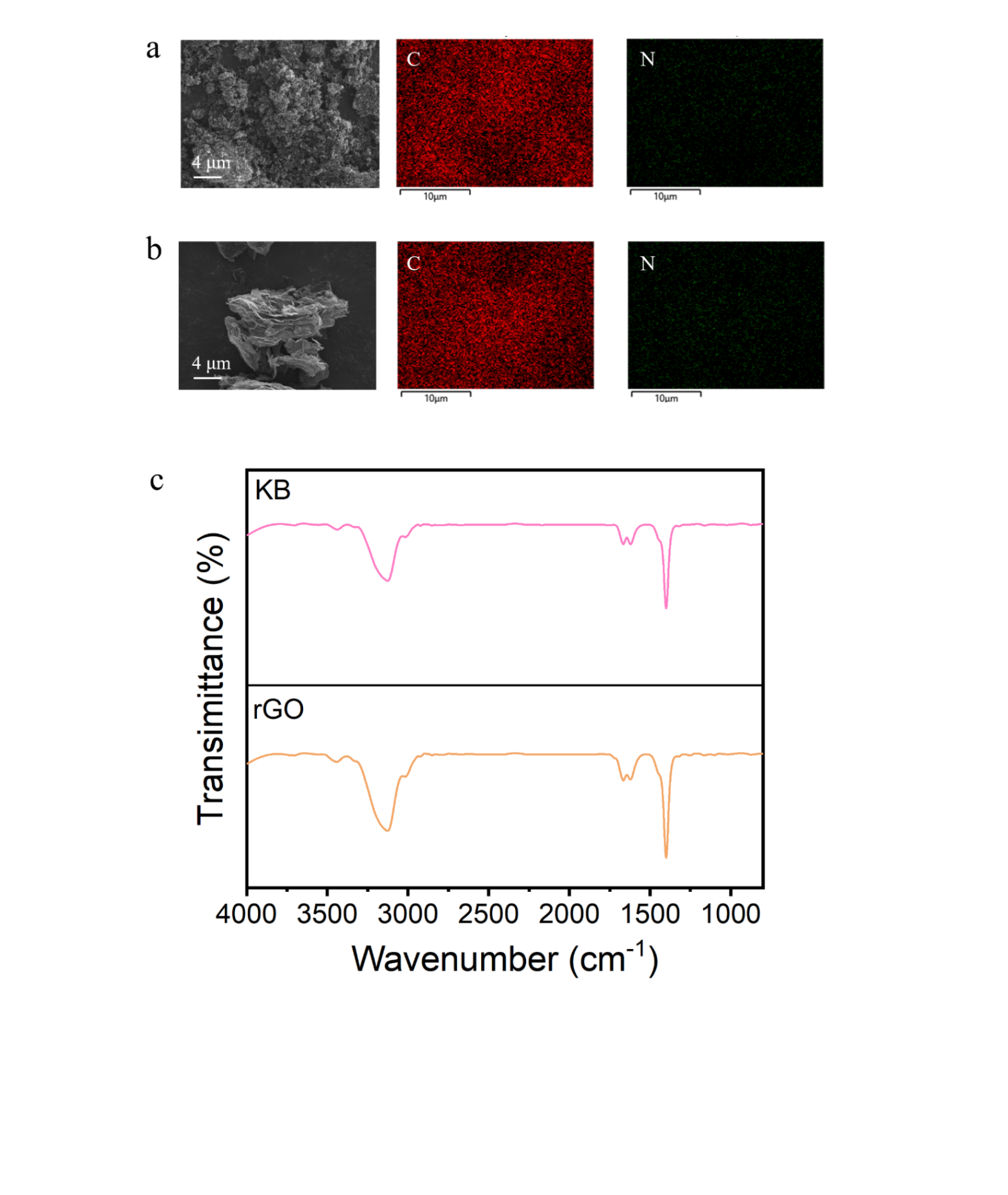

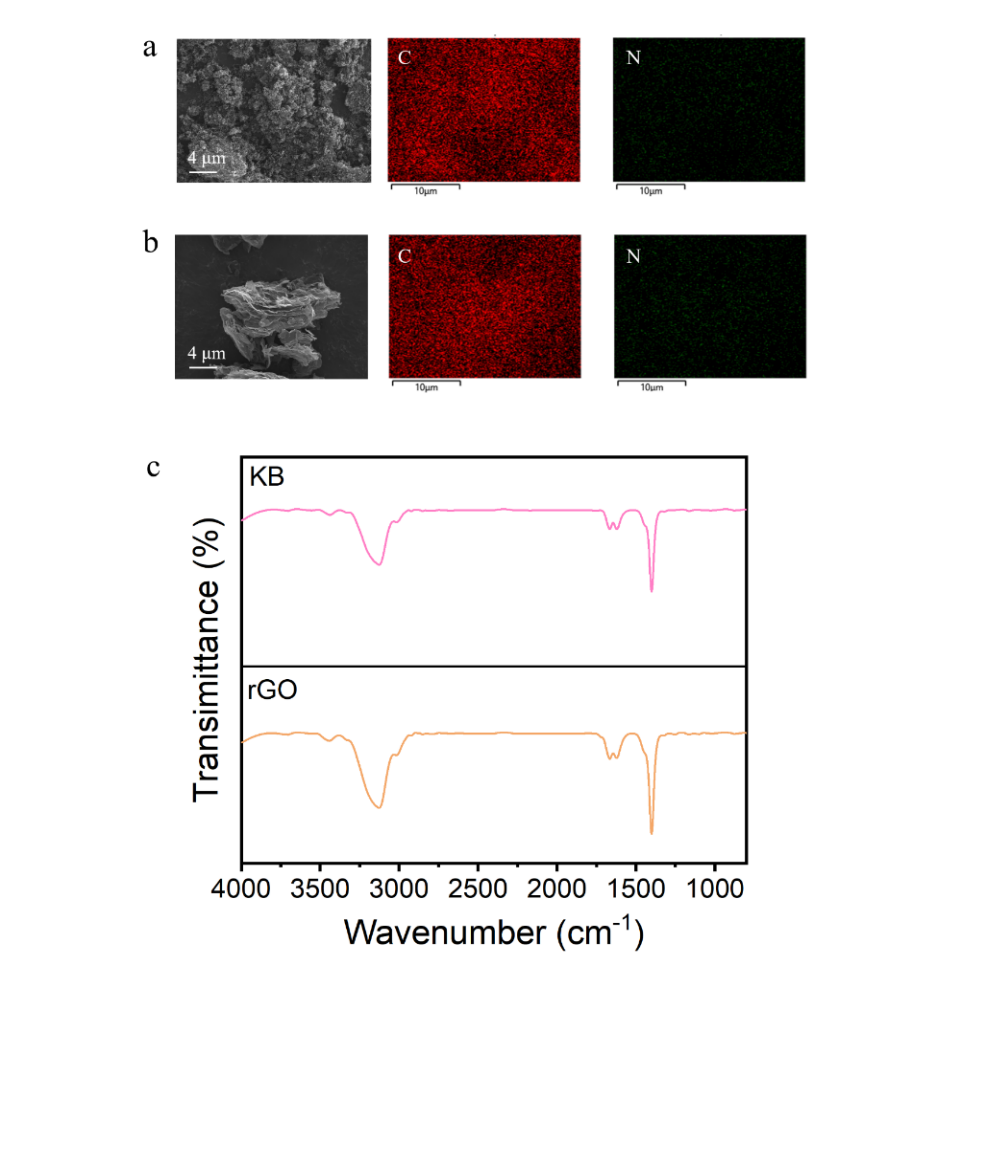


**Fig. S5** SEM-EDS mapping of (**a**) KB and (**b**) rGO, and (**c**) the FTIR spectra of KB and rGO


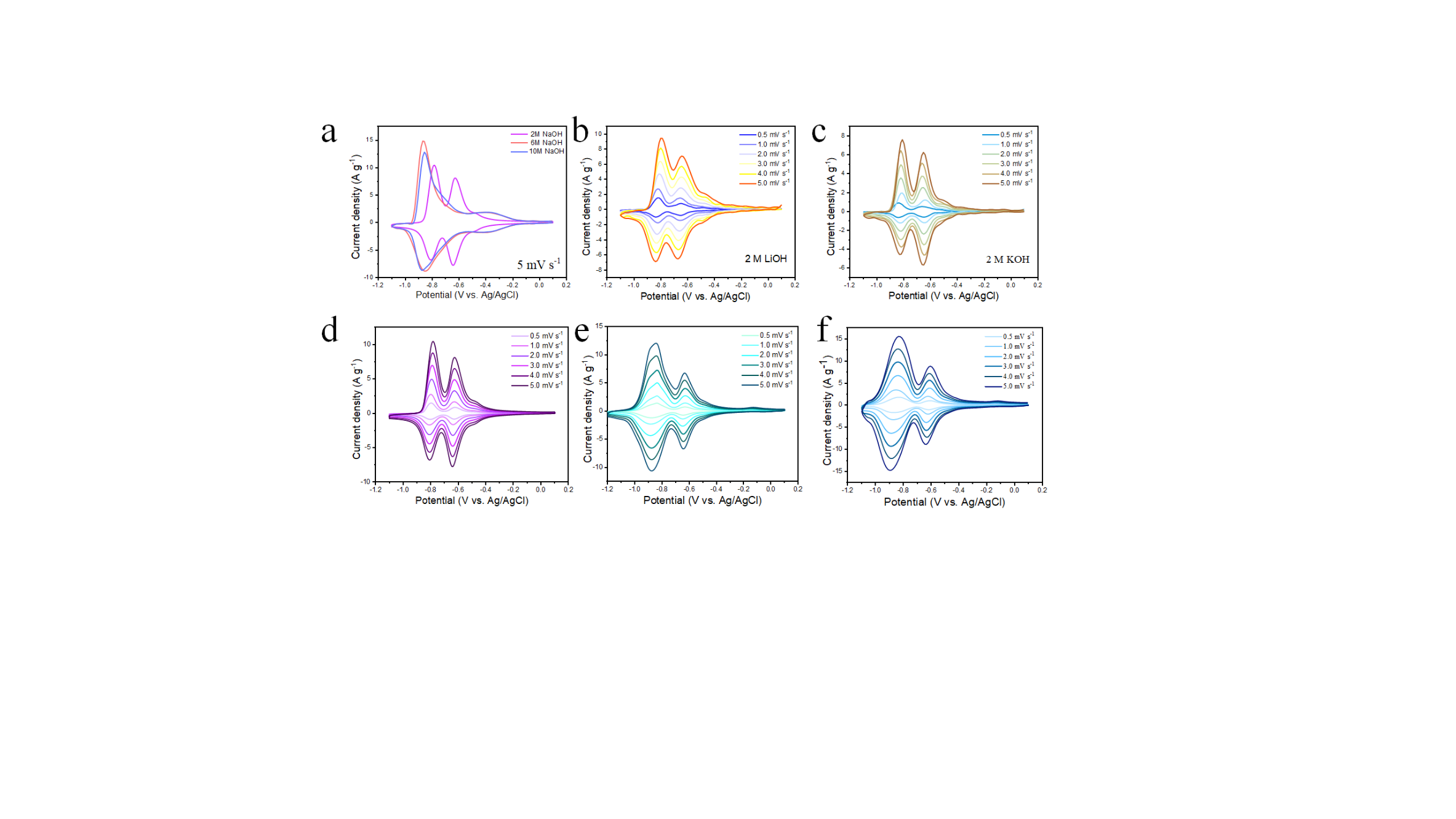


**Fig. S6** (**a**) CV curves of C_4_N at 5 mV/s for different concentrations of NaOH electrolytes. CV profiles at different scan rates of the C_4_N in (**b**) 2 M LiOH and (**c**) 2 M KOH electrolyte. CV curves of the (**d**) C_4_N, (**e**) C_4_N/KB_0.3_, and (**f**) C_4_N/KB_0.45_ electrodes at different scan rates in 2 M NaOH electrolyte


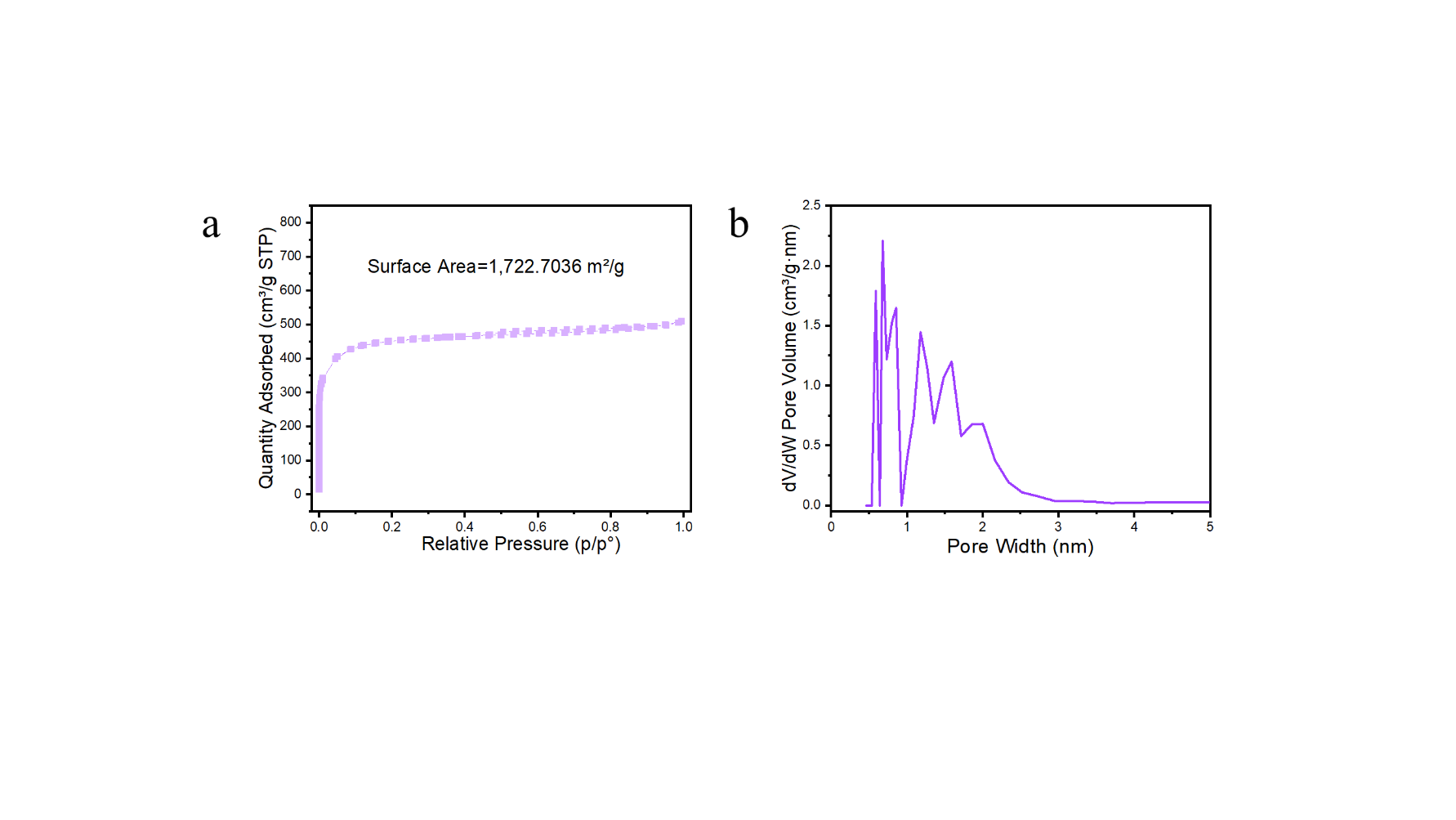


**Fig. S7** (**a**) Specific surface area and (**b**) pore size of activated carbon


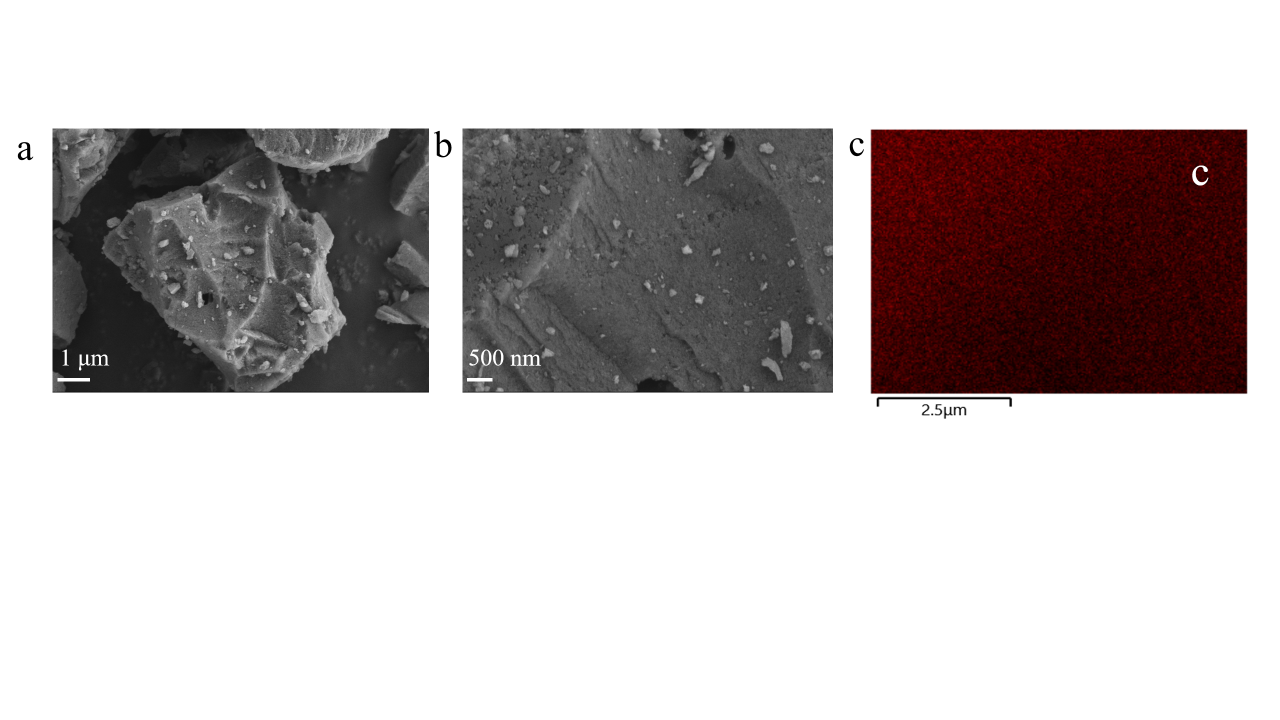


**Fig.** **S8** SEM images and EDS mapping images of activated carbon


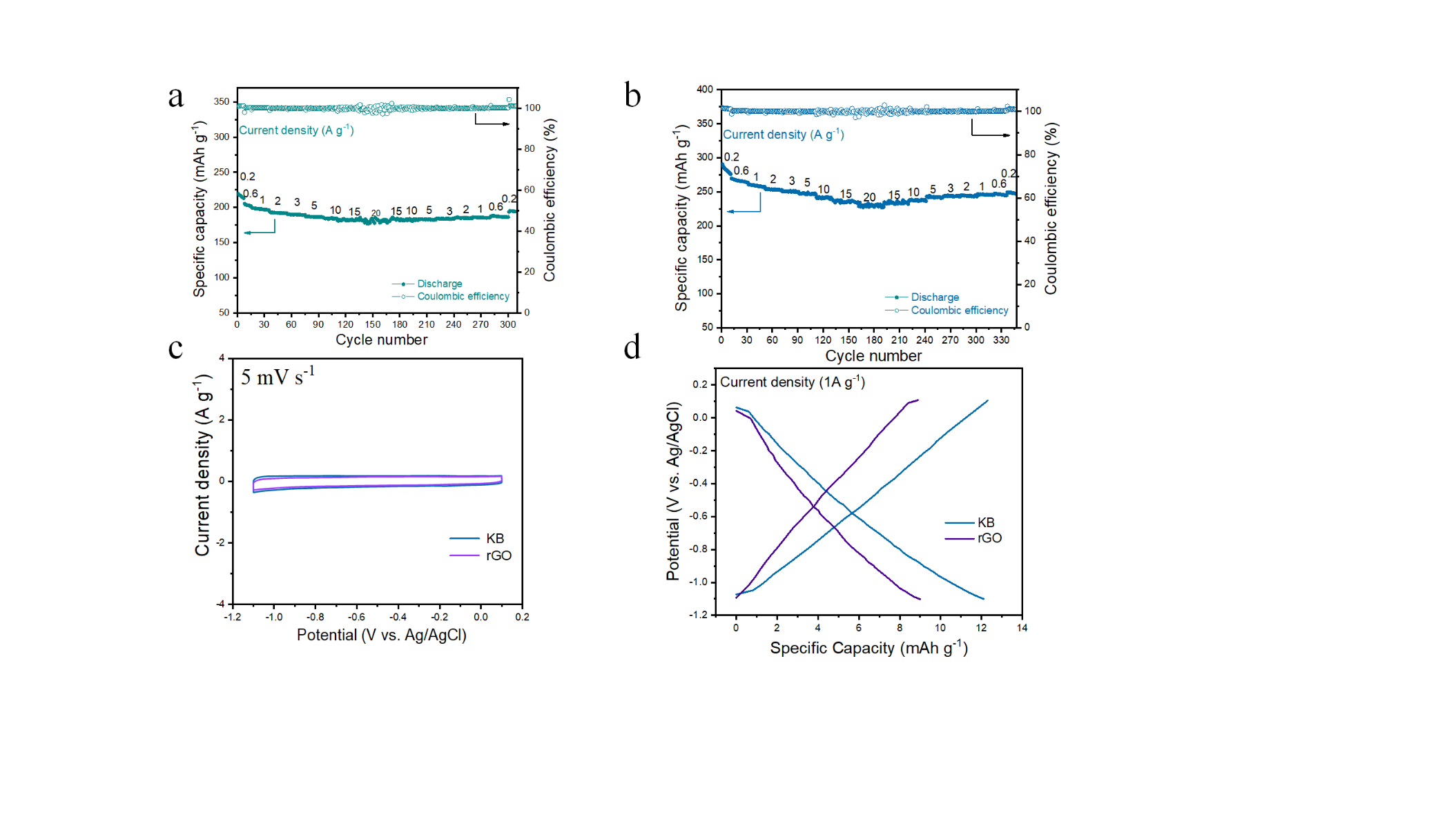


**Fig. S****9** Rate performances of the (**a**) C_4_N/KB_0.3_ and (**b**) C_4_N/KB_0.45_ in 2 M NaOH electrolyte. (**c**) CV curves and (**d**) GCD profiles of KB and rGO in 2 M NaOH electrolyte


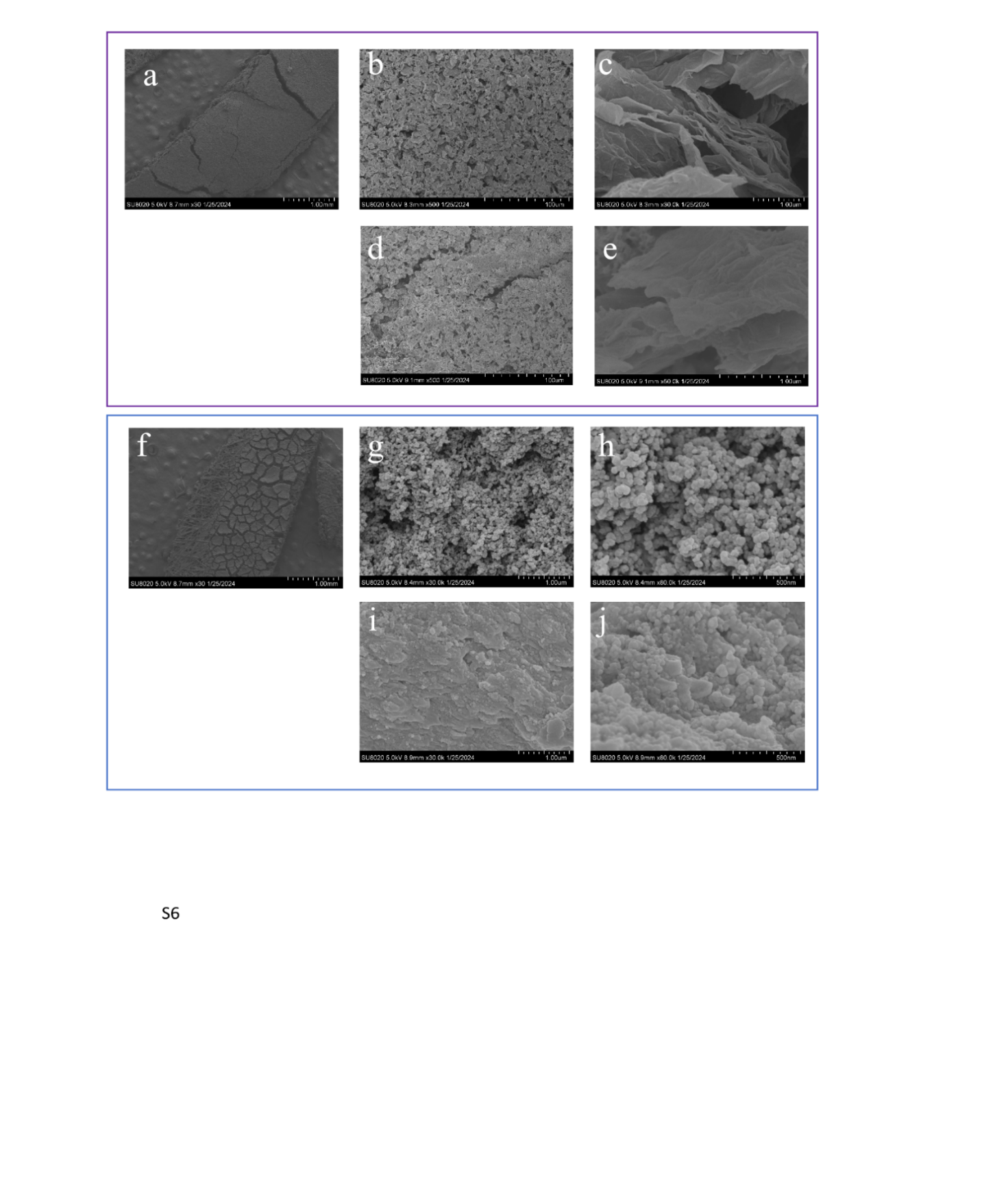


**Fig. S10** SEM images of C_4_N/rGO_0.45_ electrode sheets at different magnifications before electrochemical reaction: (**a**) 30, (**b**) 500 and (**c**) 30.0K. SEM images of C_4_N/rGO_0.45_ electrode sheets at different magnifications after electrochemical reaction: (**d**) 500 and (**e**) 50.0K. SEM images of C_4_N/KB_0.45_ electrode sheets at different magnifications before electrochemical reaction: (**f**) 30, (**g**) 30.0K and (**h**) 80.0K. SEM images of C_4_N/KB_0.45_ electrode sheets at different magnifications after electrochemical reaction: (**i**) 30.0K and (**j**) 80.0K

**
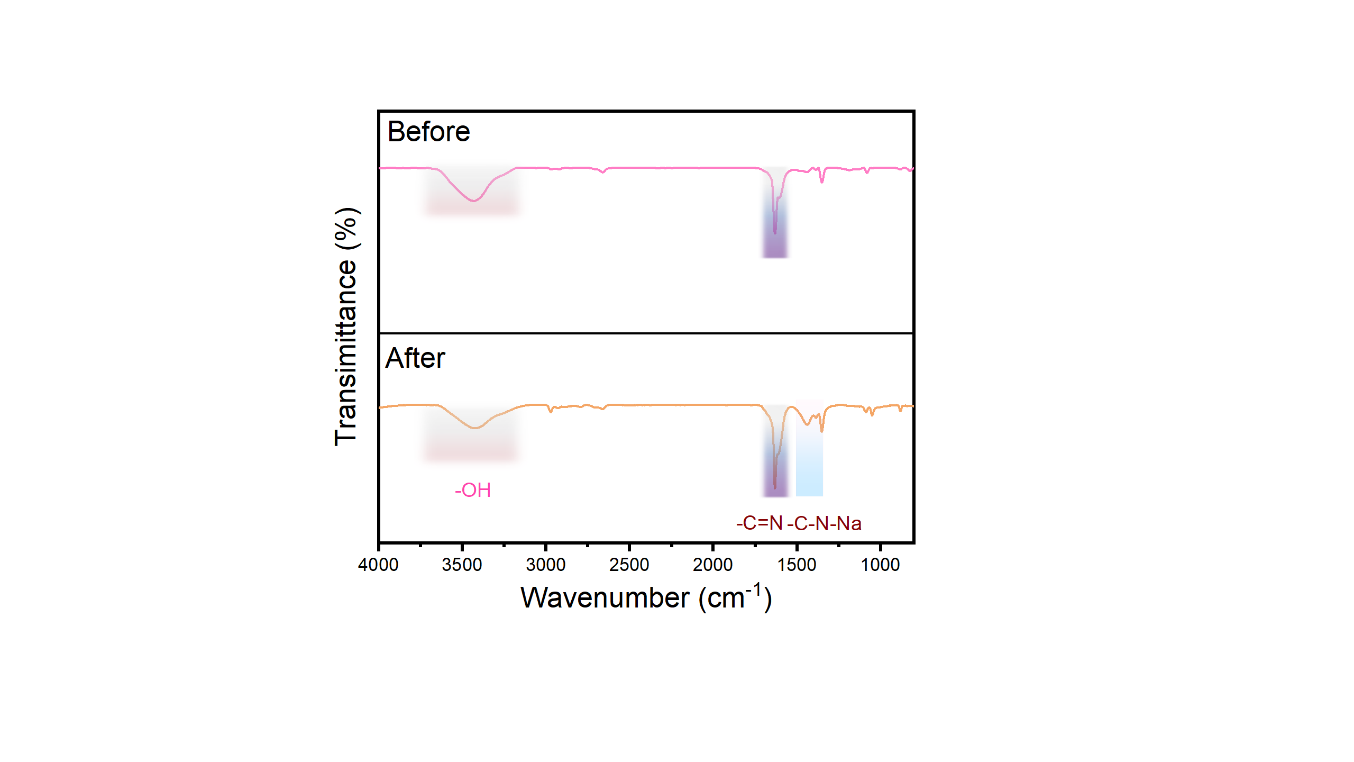
**

**Fig. S11** FT-IR of C_4_N/rGO_0.45_ electrode materials at different magnifications before and after electrochemical reaction


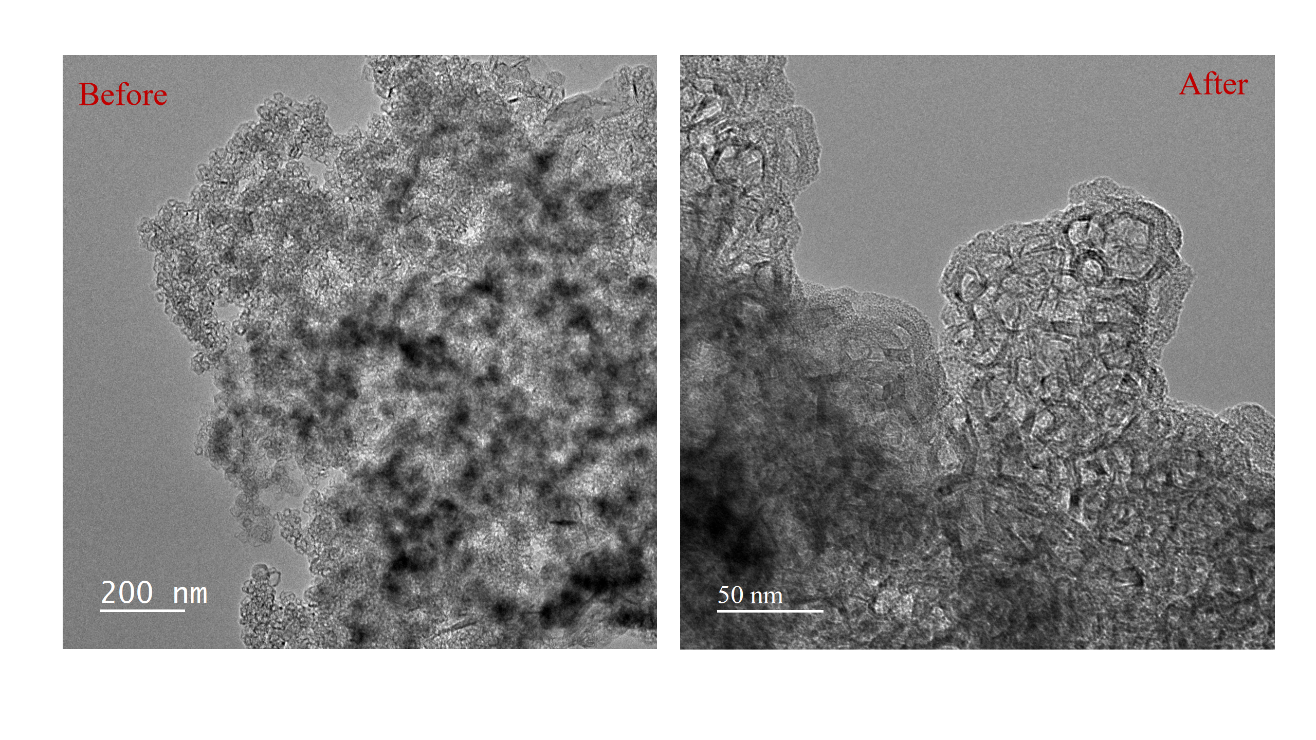


**Fig. S12** TEM images of C_4_N/rGO_0.45_ electrode materials at different magnifications before and after electrochemical reaction

**
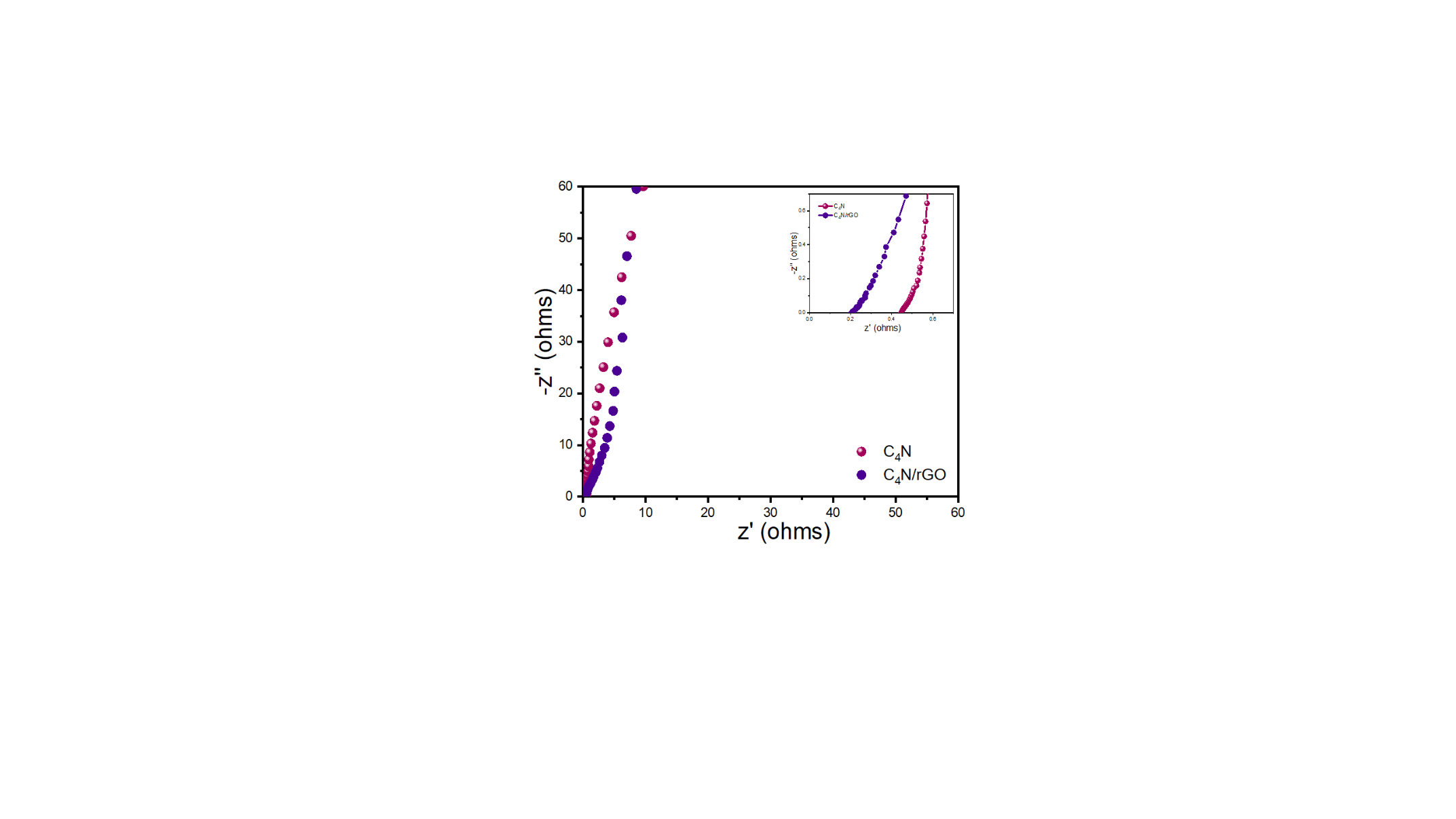
**

**Fig. S13** EIS plots of C_4_N and C_4_N/rGO_0.45_ electrodes


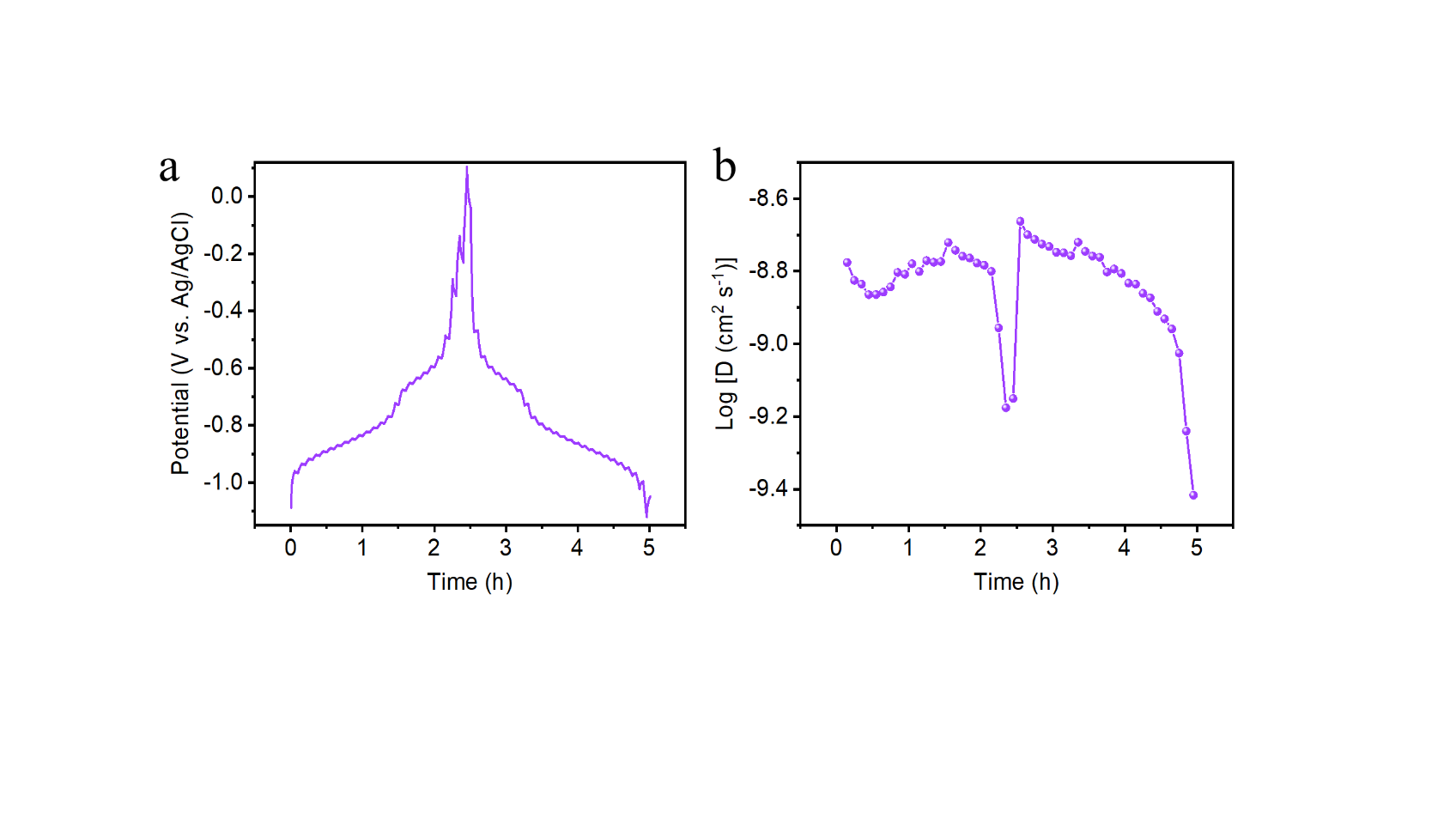


**Fig. S14** (**a**) GITT curve and (**b**) ion diffusion coefficient of C_4_N/rGO

**
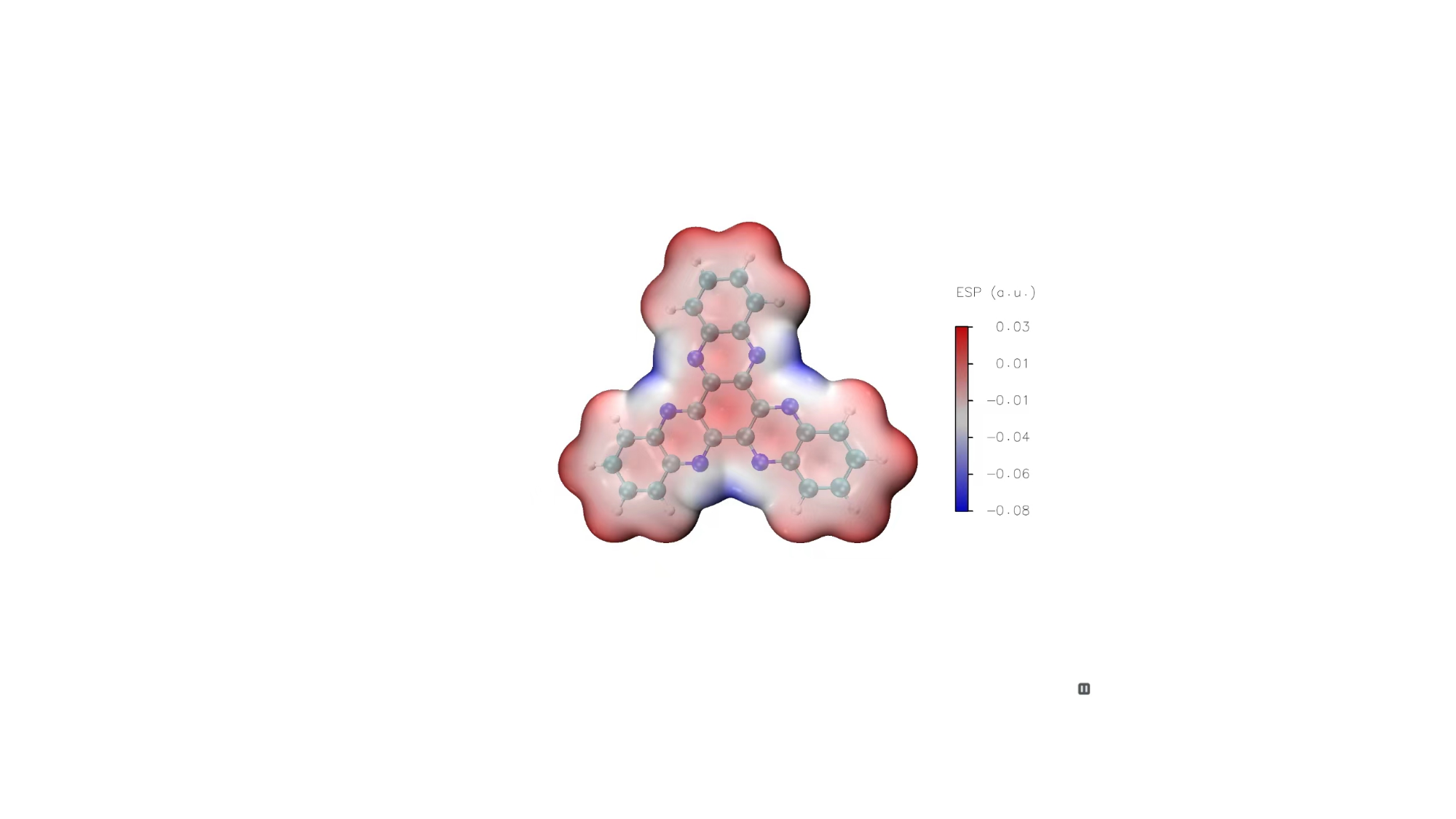
**

**Fig. S15** MESP of the monomer of C_4_N

**
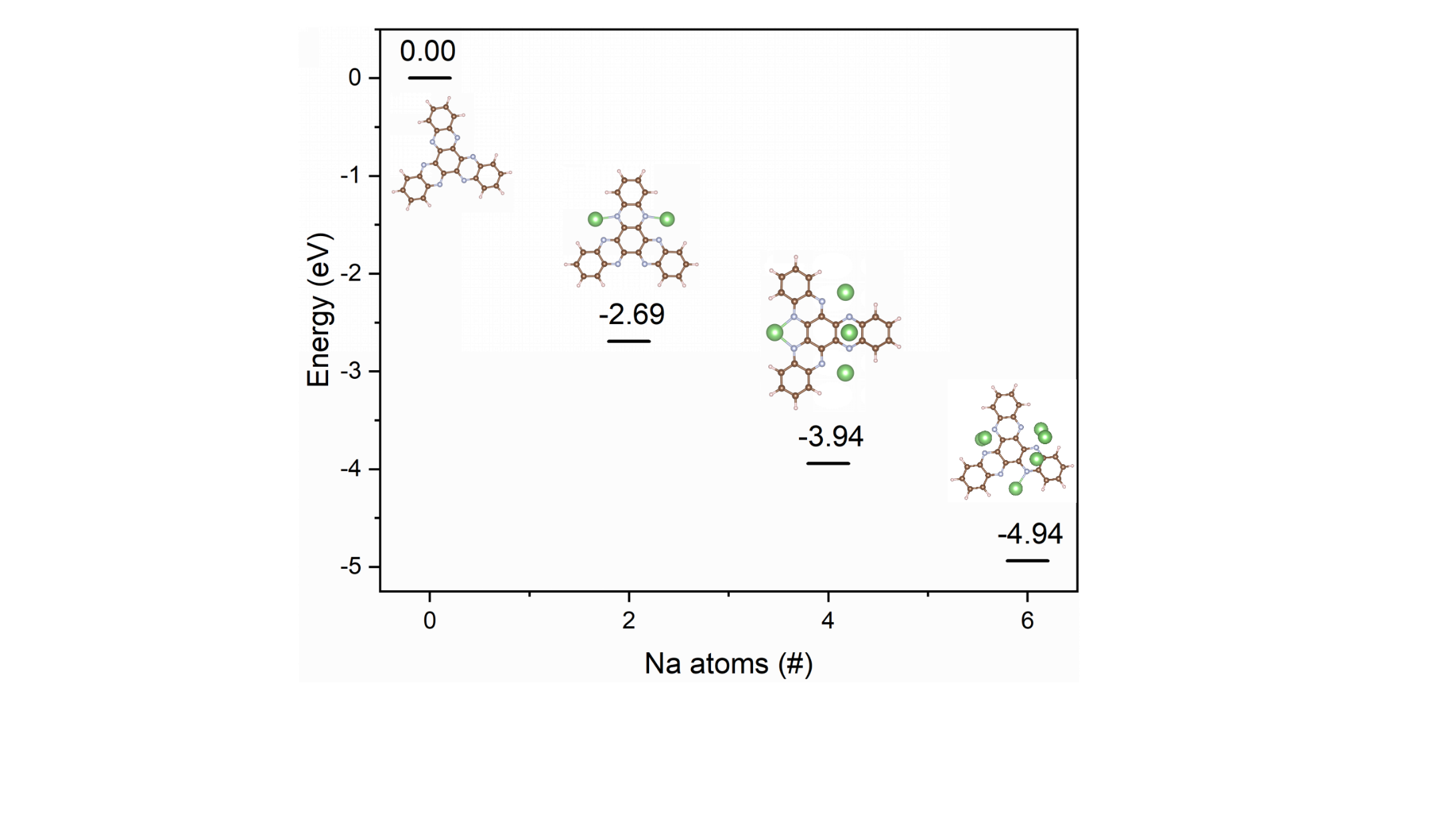
**

**Fig. S16** The free energy diagram of Na incorporation into the C_4_N


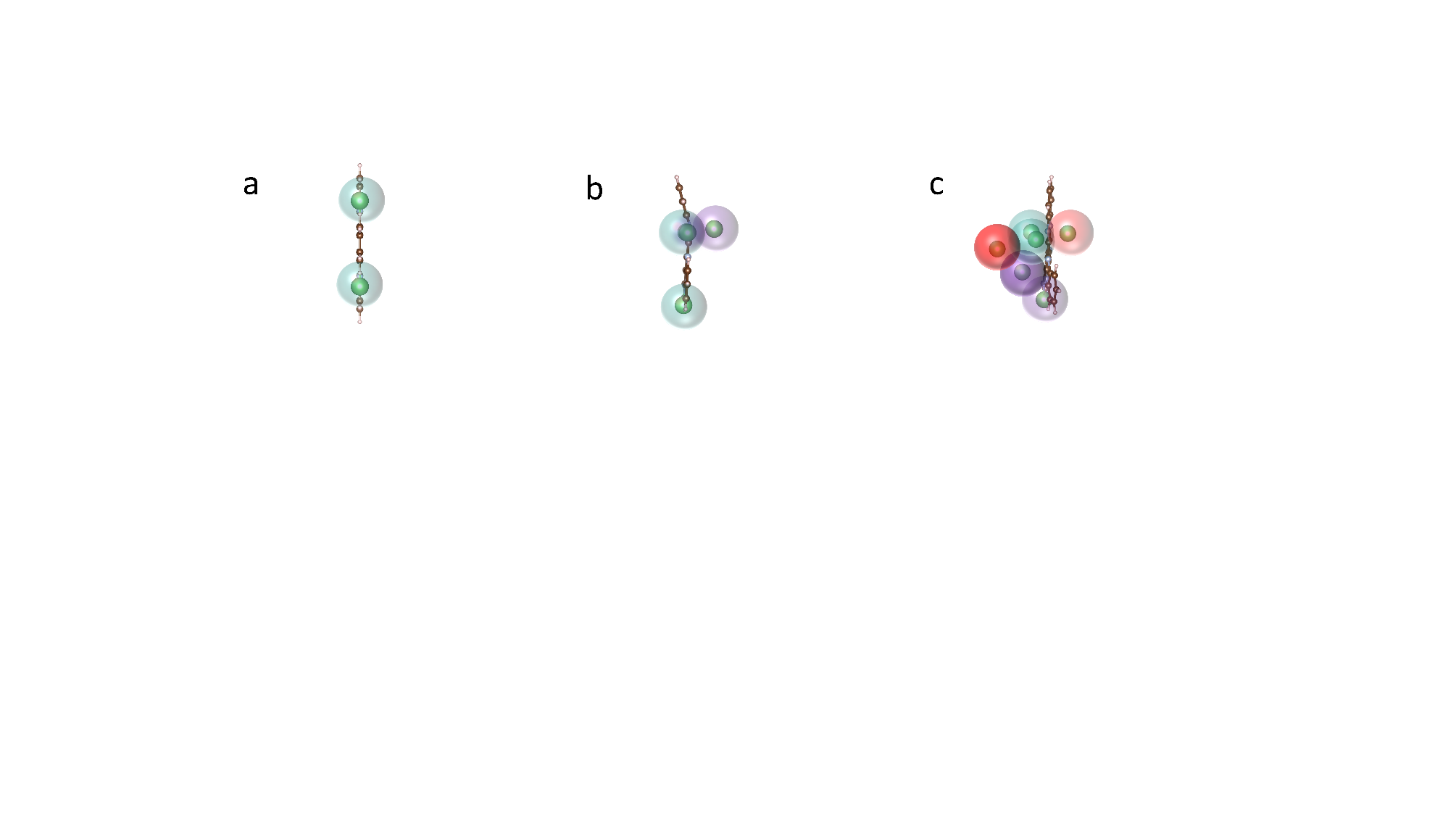


**Fig. S17** Side Schematic of the optimized structures for the sodiation of C_4_N compounds


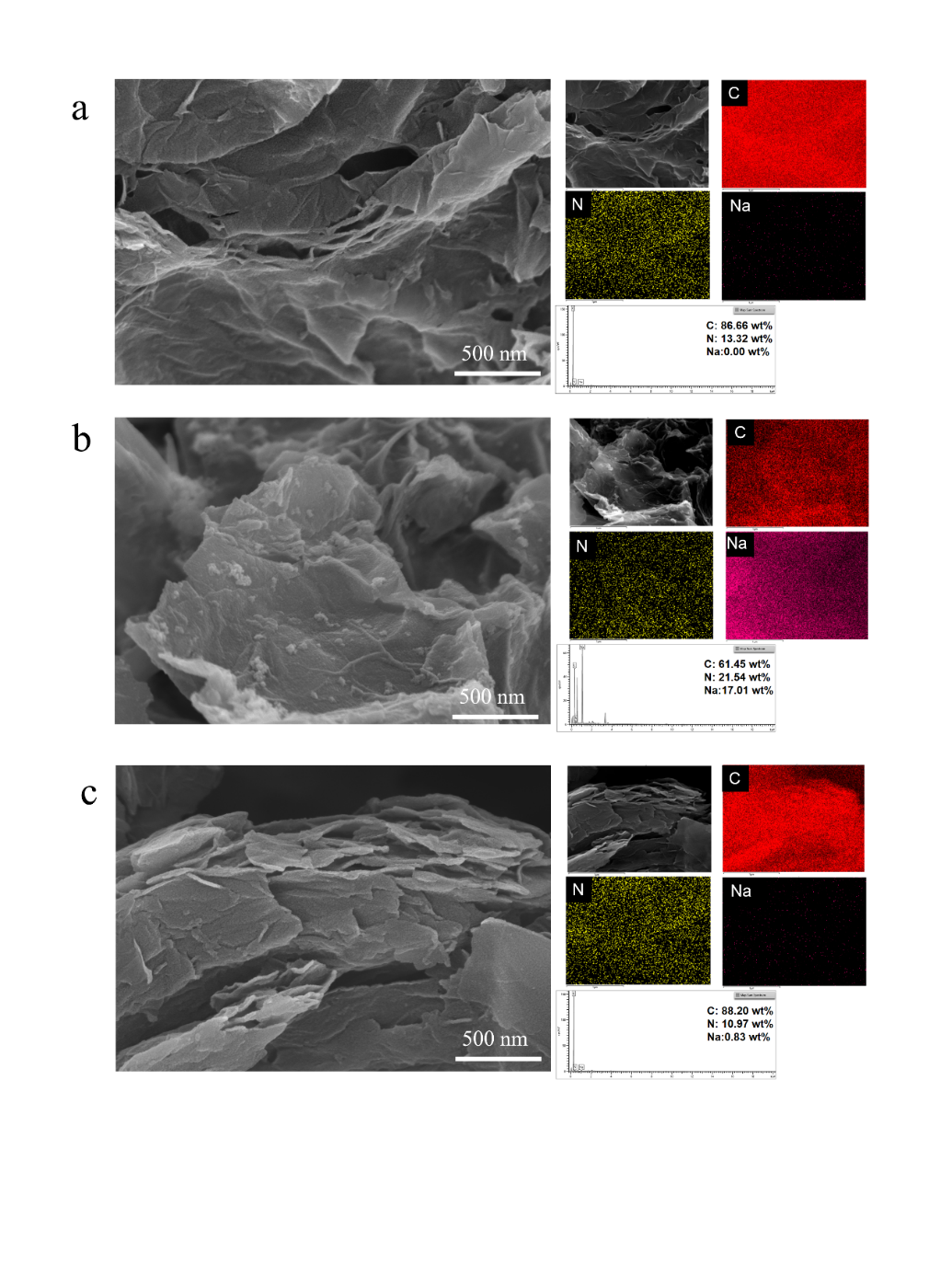


**Fig. S18** SEM, EDS mapping and elemental content of C_4_N/rGO material at different potentials: (**a**) 0.1 V, (**b**) -1.1 V and (**c**) 0 V during the charge/discharge process


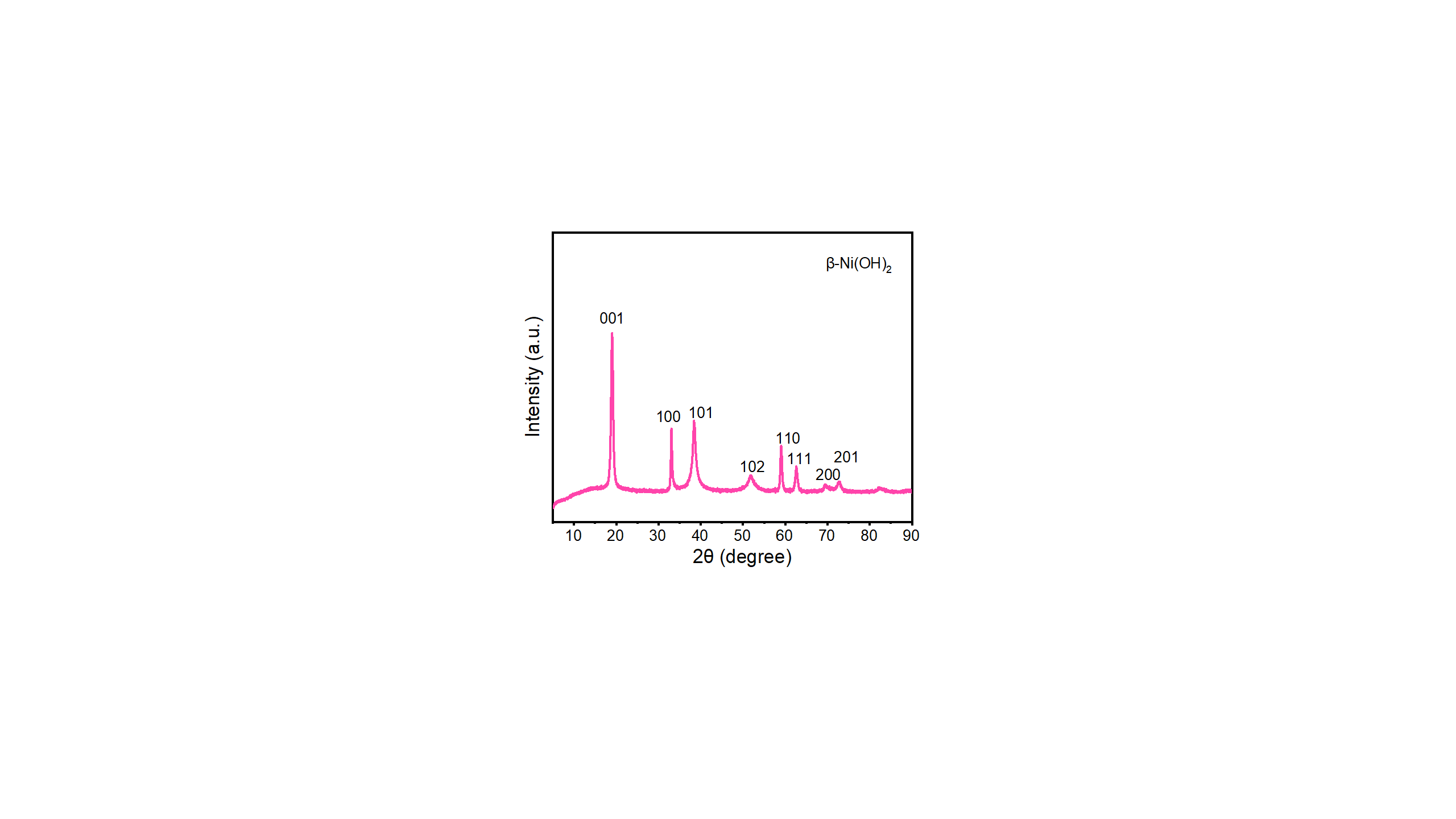


**Fig. S19** X-ray diffraction pattern of Ni(OH)_2_


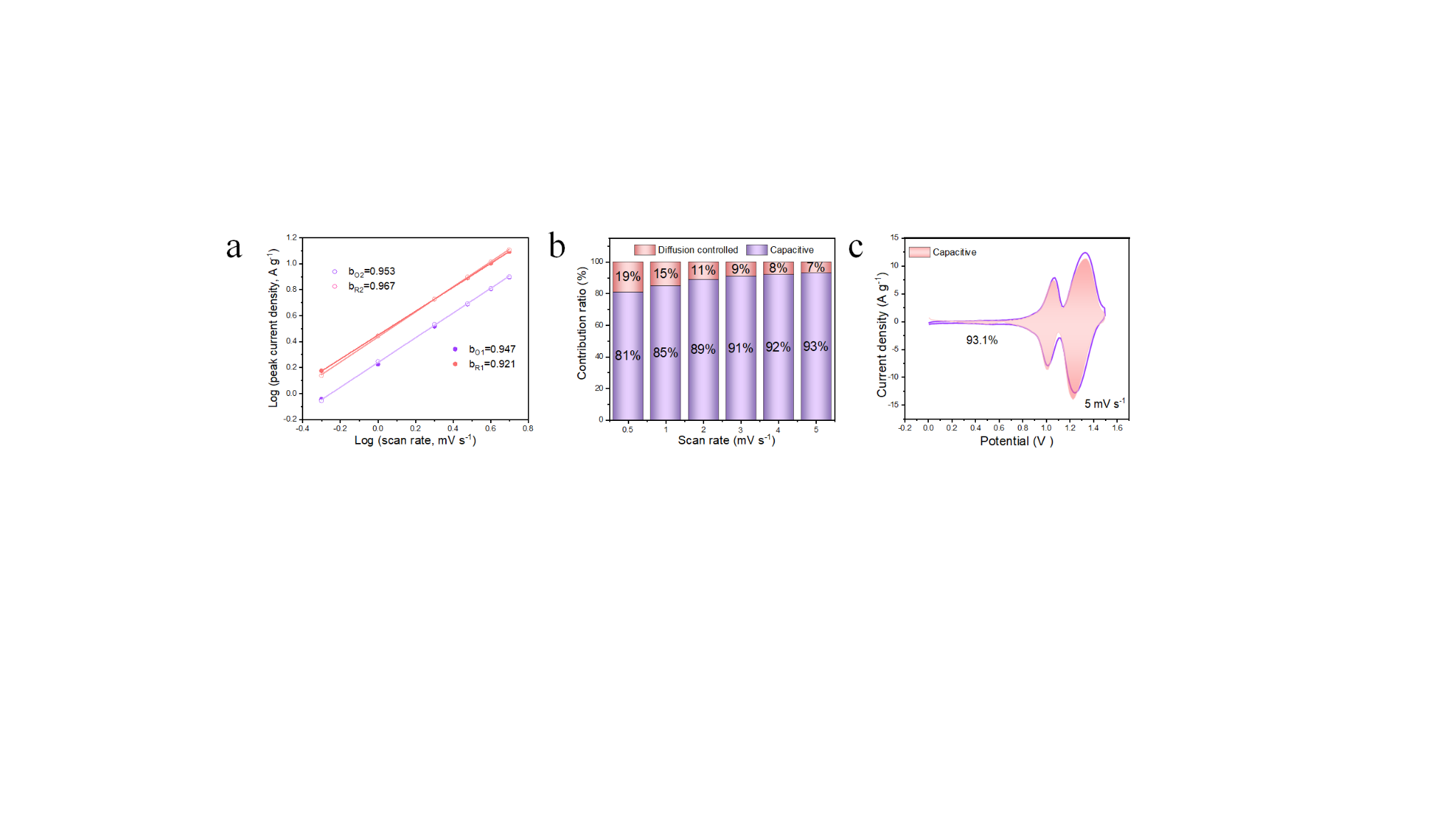


**Fig. S20** Reaction kinetics of the full battery in 2 M NaOH. (**a**) Linear logarithm relationship between the peak current densities and scan rates. (**b**) Contribution of the capacitive type capacity to the total capacity. (**b**) Capacitive contribution at 5 mV s^-1^


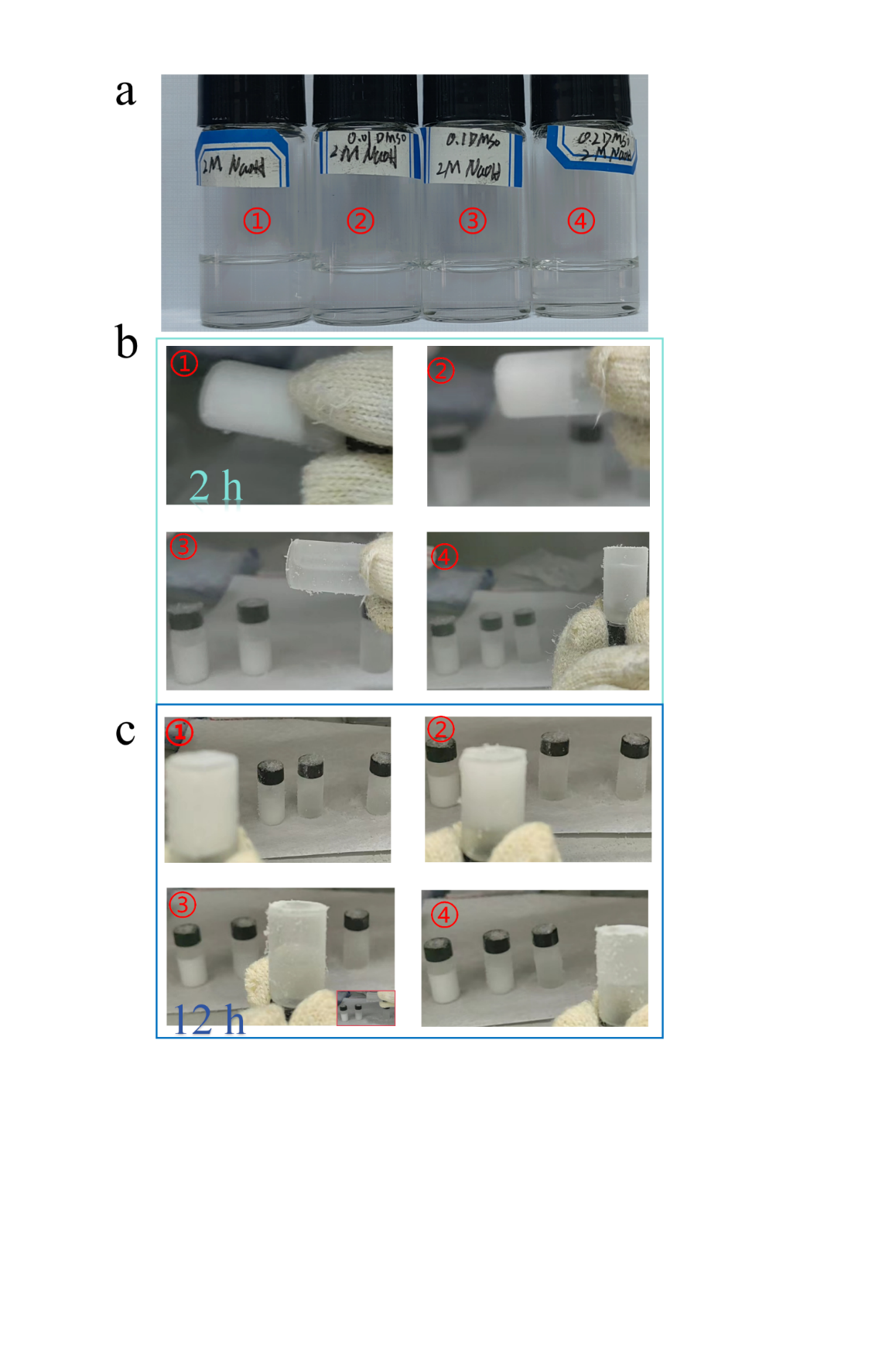


**Fig. S21** The optical photographs of different electrolytes with different molar fractions of DMSO (0, 0.01, 0.1 and 0.2) cooled at -70 °C for (**a**) 0, (**b**) 2 and (**c**) 12 h


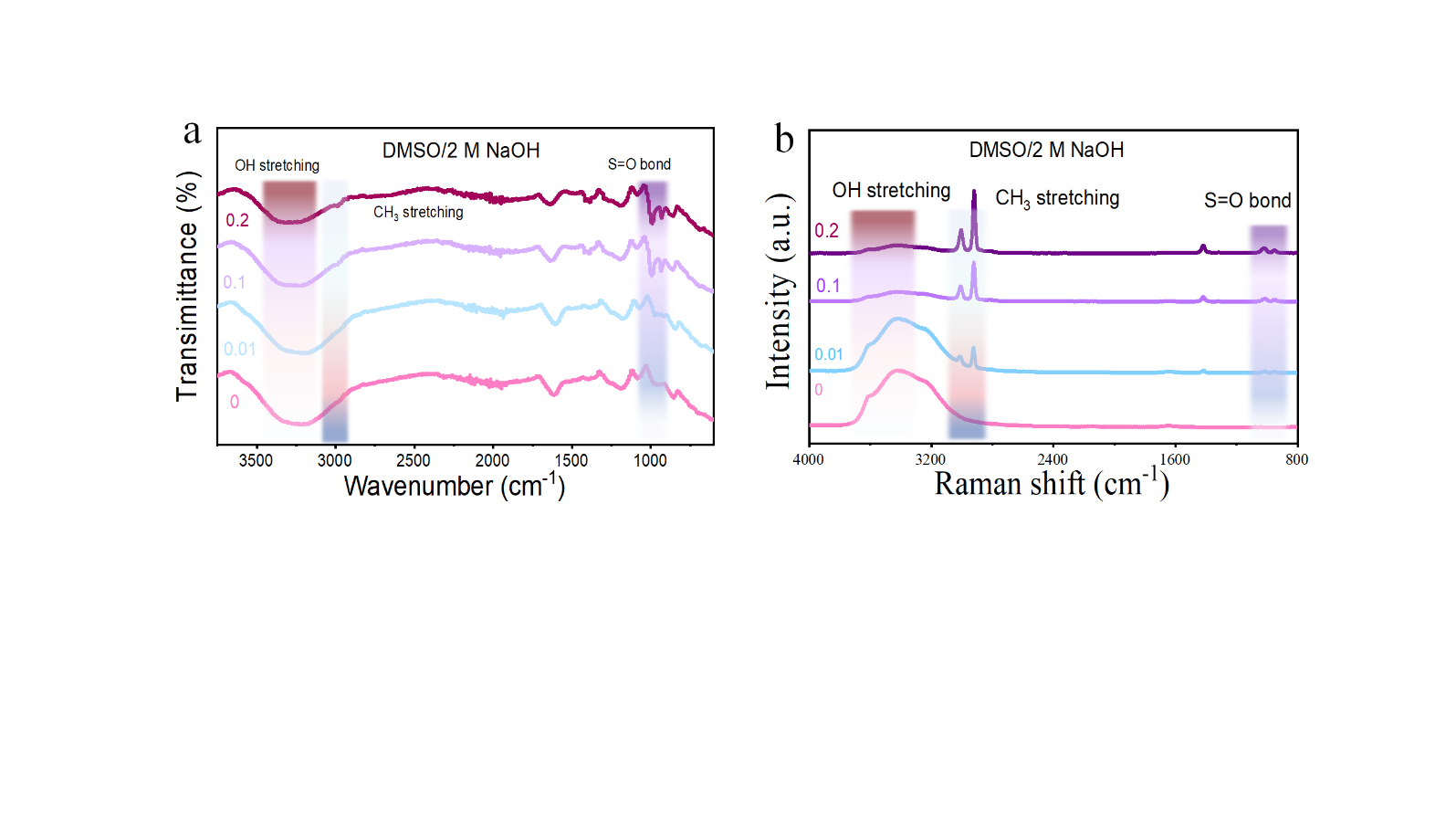


**Fig. S22** (**a**) The full FTIR spectra and (**b**) the full Raman spectra for different electrolytes with different molar fraction of DMSO from 0 to 0.2


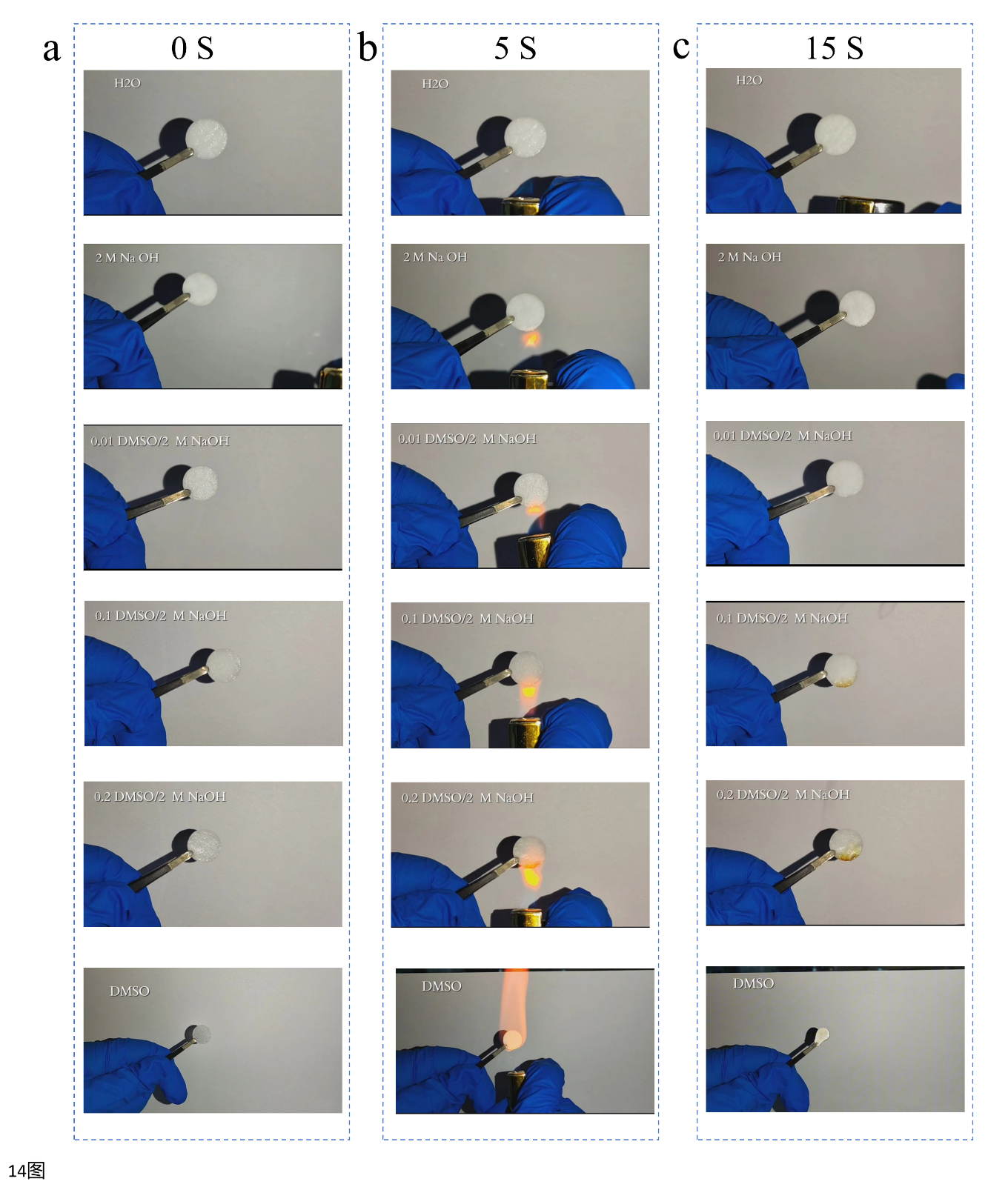


**Fig. S23** Optical pictures of different electrolytes burning for different durations (**a**) 0 seconds, (**b**) 5 seconds and (**c**) 15 seconds


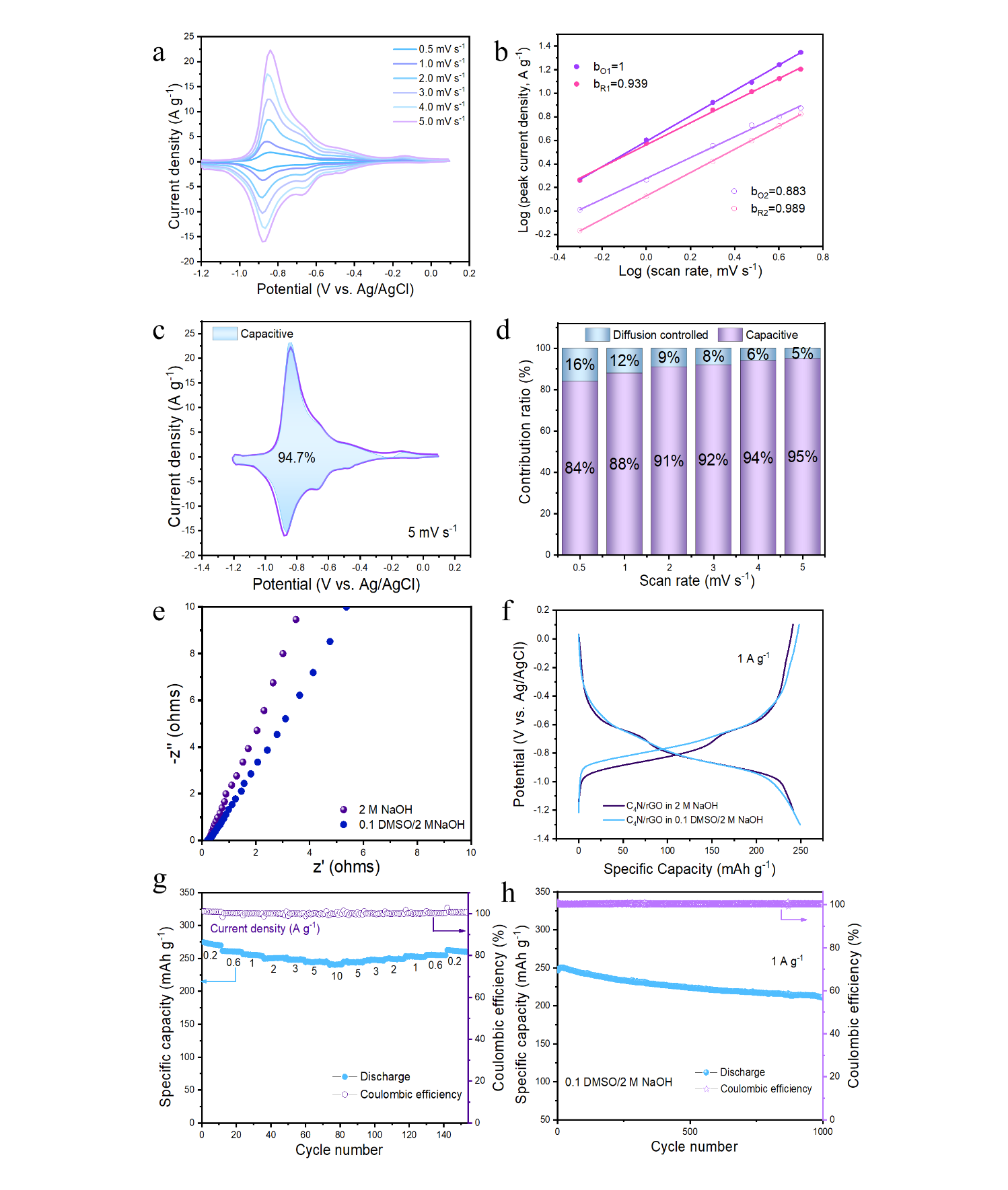


**Fig. S****24** Electrochemical properties and reaction kinetics of the C_4_N/rGO electrode in 0.1 DMSO/2 M NaOH. (**a**) CV profiles at different scan rates. (**b**) Linear logarithm relationship between the peak current densities and scan rates. (**c**) Capacitive contribution at 5 mV s^-1^. (**d**) The ratios of capacitive contribution to the total capacity at different scan rates. (**e**) The Nyquist plots and (**f**) charge/discharge profiles of C_4_N/rGO electrode in 2 M NaOH and 0.1 DMSO/2 M NaOH electrolytes. (**g**) Rate performance and (**h**) cycle stability at 1 A g^-1^ of the C4N/rGO electrode in 0.1 DMSO/2 M NaOH


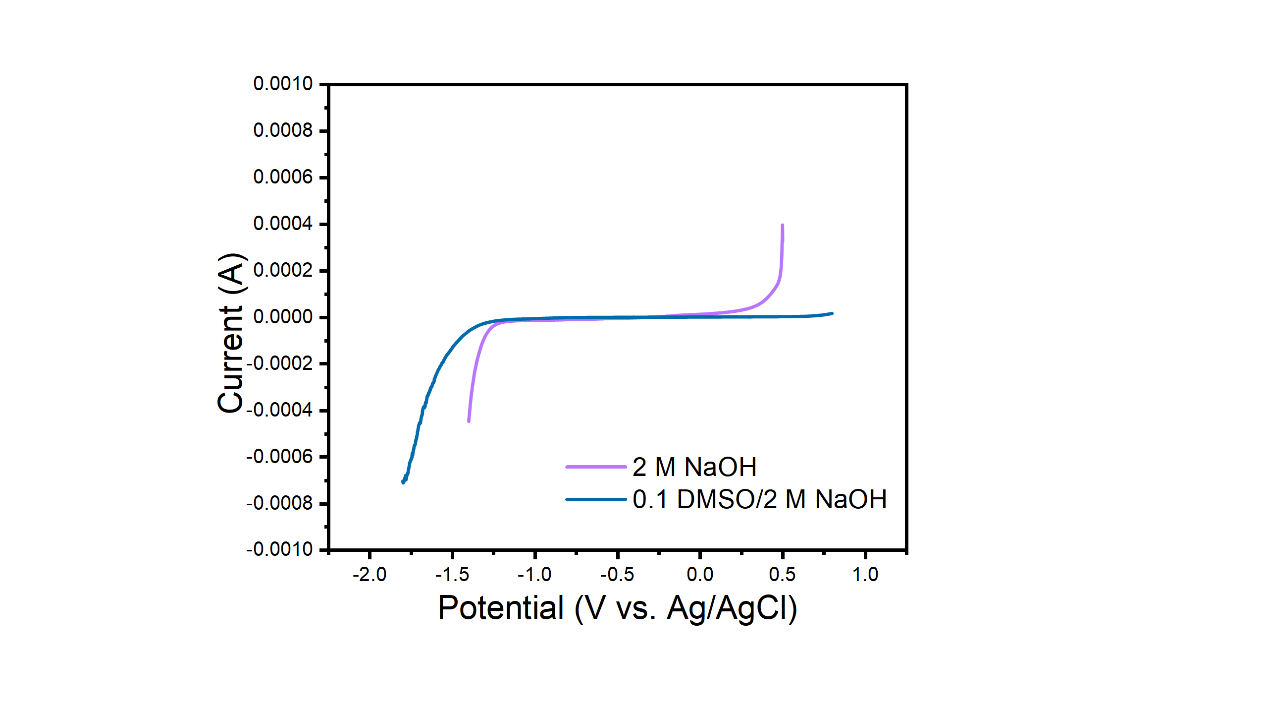


**Fig. S25** Linear scanning voltammetry curves (LSV) of different electrolytes tested at 1 mV s^-1^ through two titanium foils electrodes


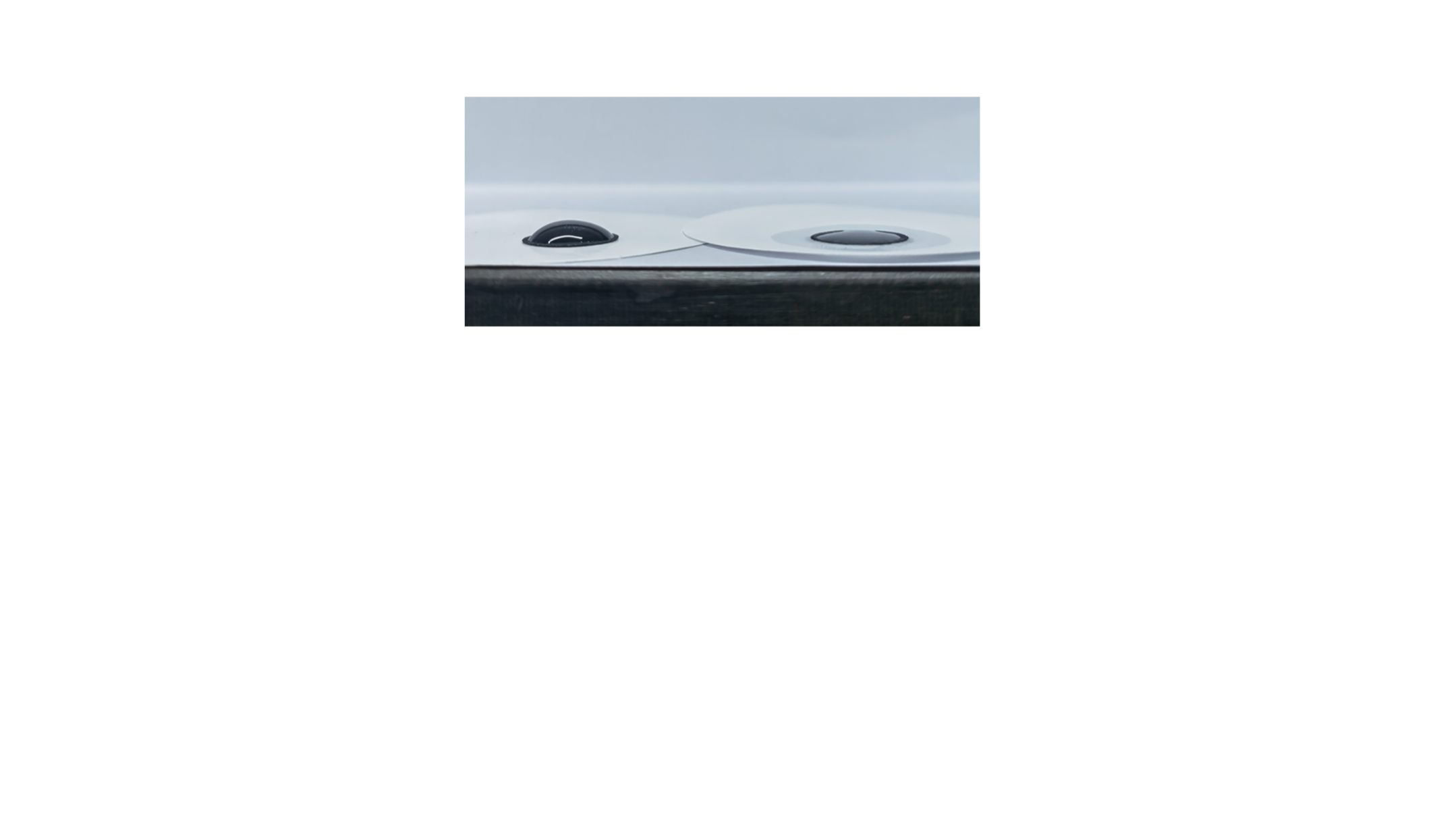


**Fig. S26** Wettability experiments: C_4_N/rGO_0.45_ electrode sheet with drops of electrolyte 2 M NaOH (left) and 0.1 DMSO/2 M NaOH (right)


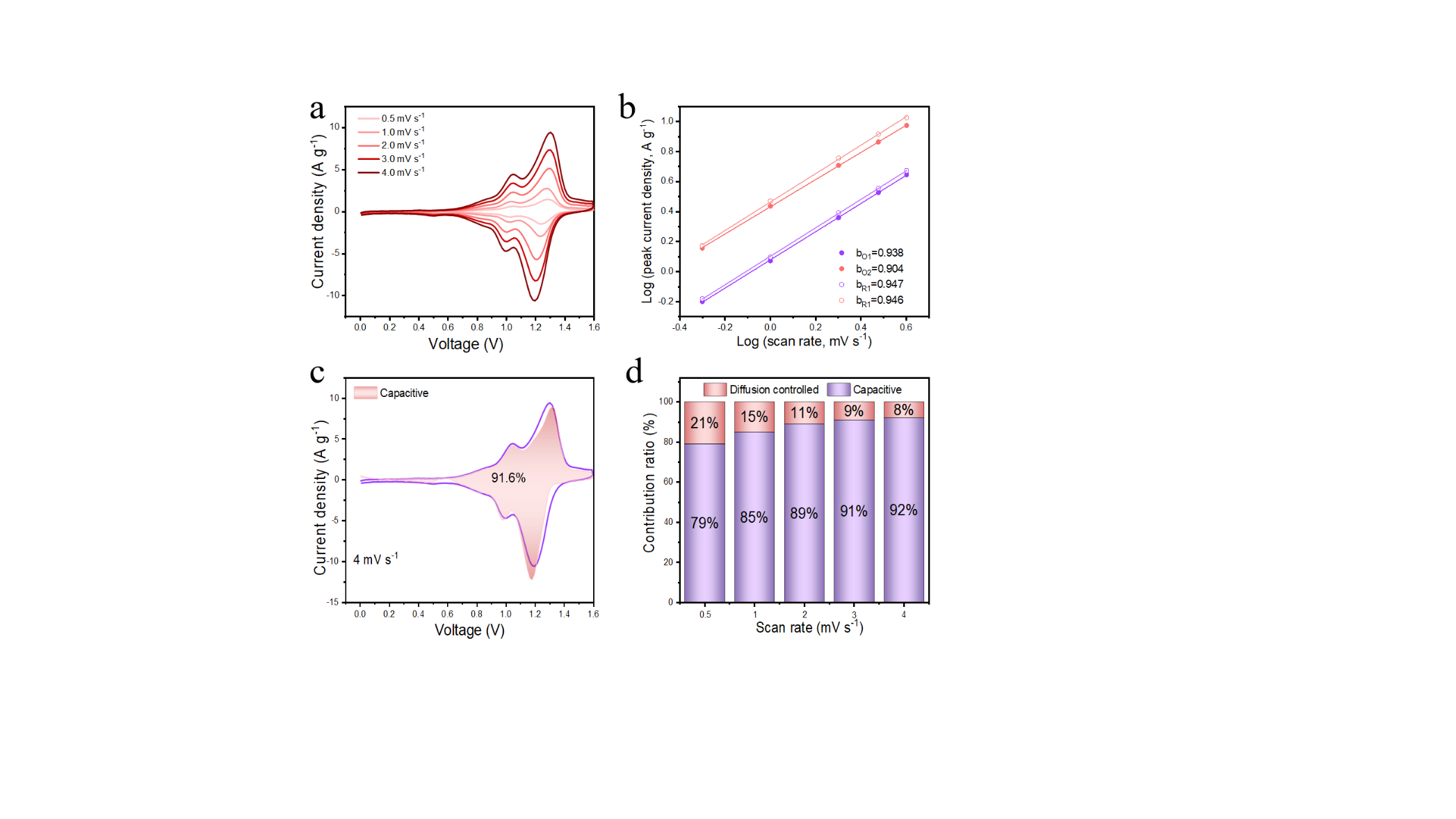


**Fig. S27** Reaction kinetics evaluation of the full battery in 0.1 DMSO/2 M NaOH. (**a**) CV profiles at different scan rates. (**b**) Linear logarithm relationship between the peak current densities and scan rates. (**c**) capacitive contribution at 4 mV s^-1^. (**d**) The ratios of capacitive contribution to the total capacity at different scan rates


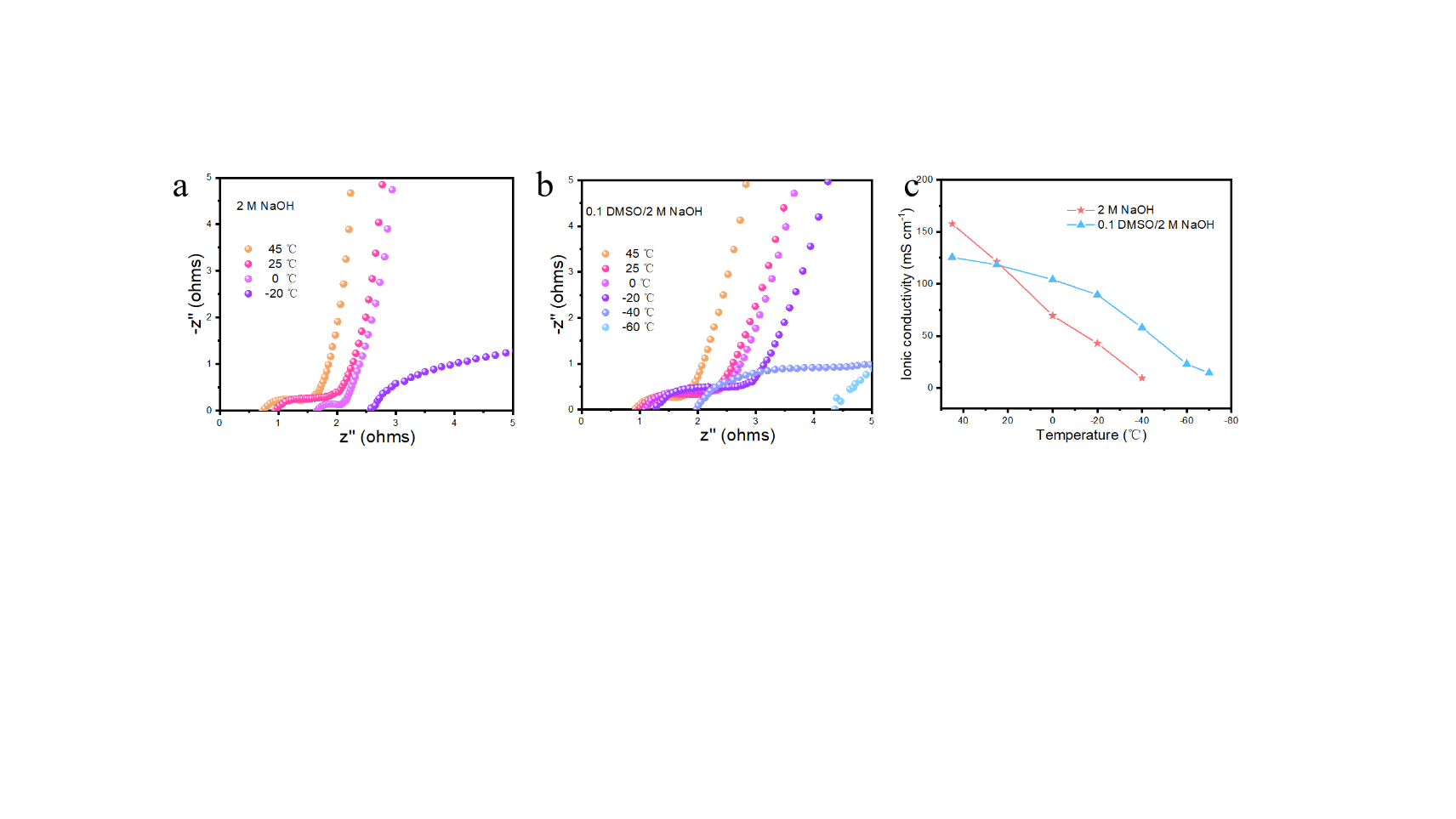


**Fig. S28** Nyquist plots of full cells with different electrolytes. (**a**) 2 M NaOH and (**b**) 0.1 DMSO/2 M NaOH. (**c**) The temperature-dependent ionic conductivity

**
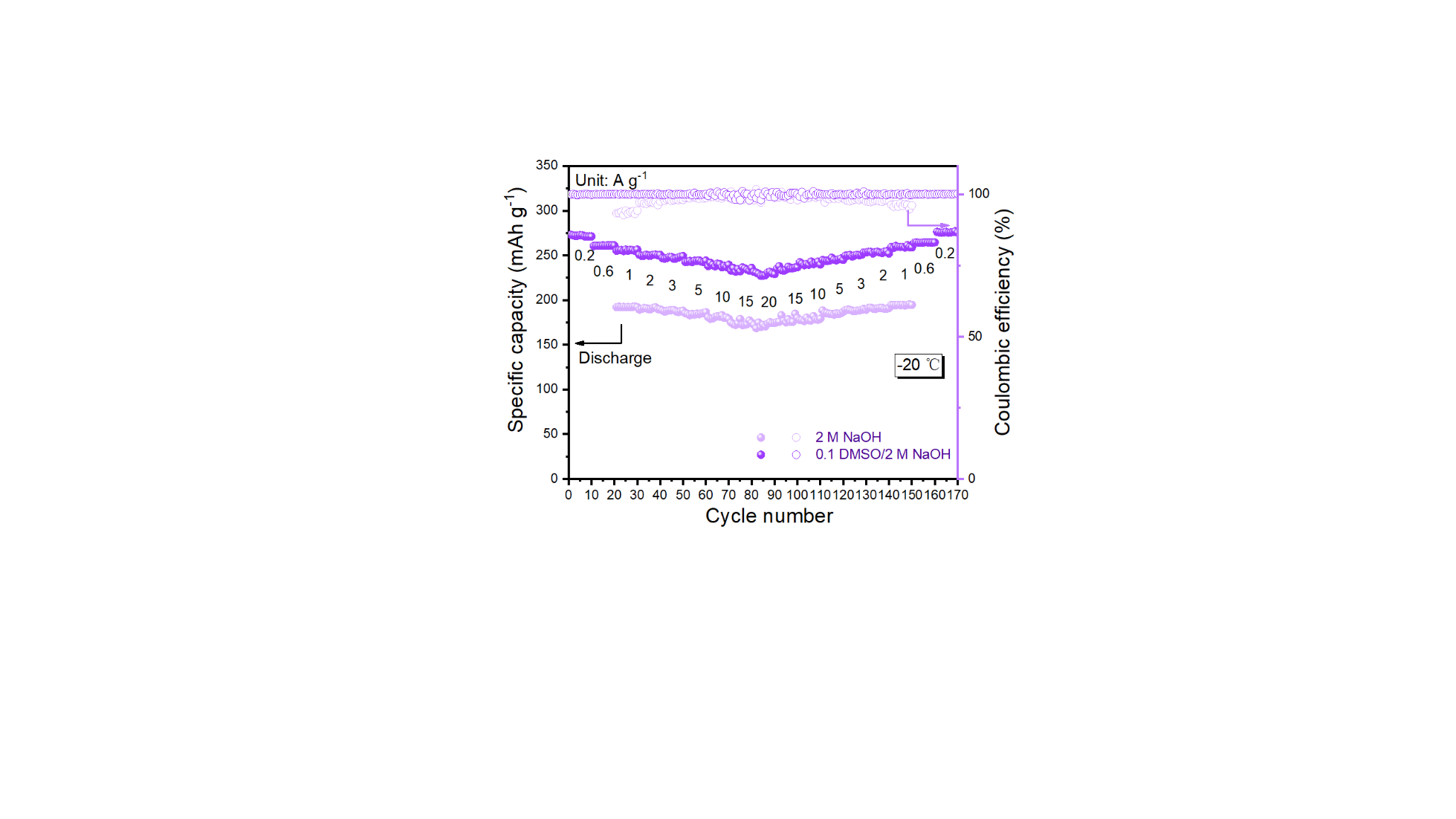
**

**Fig. S29** Rate performance of the full cell with different electrolytes at -20 ℃

**Table S1** Organic elemental analysis of C_4_N

| **Element** | C | H | N |
| --- | --- | --- | --- |
| **Weight (%)** | 62.757 | 3.729 | 18.25 |
| **Mass ratio of C/N** |  | 3.44:1 |  |
| **Molar atomic ratio of C/N** |  | 4.01:1 |  |

**Table S2** Electronic conductivity of different anode materials

| **Sample** | **1MPa** | **2MPa** | **3MPa** | **Units** |
| --- | --- | --- | --- | --- |
| C_4_N | 3.387E^-12^ | 4.174E^-12^ | 5.44E^-12^ | S·m^-1^ |
| C_4_N/KB_0.3_ | 5.616E^-05^ | 5.860E^-05^ | 6.51E^-05^ | S·m^-1^ |
| C_4_N/KB_0.45_ | 3.436E^-02^ | 5.228E^-02^ | 9.80E^-02^ | S·m^-1^ |
| C_4_N/rGO_0.45_ | 5.117E^+01^ | 1.057E^+02^ | 2.01E^+02^ | S·m^-1^ |
| KB |  | 3.462E^+02^ |  | S·m^-1^ |
| rGO |  | 1.38E^+03^ |  | S·m^-1^ |

**Table S3** Performance Comparison of Commercial Alkaline Nickel-Based Batteries

|  | **Capacity** | **Nontoxity** | **Element abundance** | **Voltage** | **Cycle number** |
| --- | --- | --- | --- | --- | --- |
| This work | 258 | 5 | 5 | 1.24 | 38,000 |
| Zn//Ni [S1] | 500 | 3 | 3 | 1.7 | 2000 |
| MmH//Ni [S2] | 300 | 4 | 1 | 1.2 | 5000 |
| Cd//Ni [S3] | 180 | 1 | 2 | 1.2 | 2000 |

**Table S4** Hydrogen bond numbers and Ewald Energy for different electrolytes

|  | Hydrogen bond numbers | Ewald Energy (kJ/mol) |
| --- | --- | --- |
| 2M NaOH | 2468 | -1.348×10^5^ |
| 2M NaOH/0.1 M DMSO | 1523 | -1.076×10^5^ |

**Table S5** Comparison of capacity and stability of acid, medium and alkaline batteries at different temperatures

| **Entry** | **Anode** | **Cathode** | **PH** | **Specific capacity**  **(mAh g^-1^)** | **Cycles**  **(N)** | **Temperature (℃)** | **Refs.** |
| --- | --- | --- | --- | --- | --- | --- | --- |
| 1 | PTO | PbO_2_ | Acid | 395 | 1500 | 25 | [S4] |
| 2 | PTO | MnO_2_@GF | Acid | 110 | 100 | -70 | [S5] |
| 3 | Li | LFP | Neutral | 150 | 160 | -10 | [S6] |
| 4 | Zn | V_2_O_5_ | Neutral | 285 | 1000 | -30 | [S7] |
| 5 | PAQs | Ni(OH)_2_ | Alkaline | 200 | 1350 | 25 | [S4] |
| 6 | P_14_AQs | Co-Ni(OH)_2_ | Alkaline | 148.7 | 50 | -30 | [S8] |
| 7 | PNZ | NMO | Alkaline | 100 | 2000 | -20 | [S9] |
| 8 | C_4_N/rGO | Ni(OH)_2_ | Alkaline | 294.6 | 60000 | 25 | This work |
| 9 | C_4_N/rGO | Ni(OH)_2_ | Alkaline | 265.7 | 12500 | -40 | This work |
| 10 | C_4_N/rGO | Ni(OH)_2_ | Alkaline | 164.6 | 160 | -70 | This work |

**Supplementary References**

1. D. Zhou, X. Guo, Q. Zhang, Y. Shi, H. Zhang et al., Nickel‐based materials for advanced rechargeable batteries. Adv. Funct. Mater. **32**(12), 2107928 (2021). <https://doi.org/10.1002/adfm.202107928>
2. L. T. Lam, R. Louey, N. P. Haigh, O. V. Lim, D. G. Vella et al., Vrla ultrabattery for high-rate partial-state-of-charge operation. J. Power Sources **174**(1), 16-29 (2007). <https://doi.org/10.1016/j.jpowsour.2007.05.047>
3. H. Cui, D. Zhang, Z. Wu, J. Zhu, P. Li et al., Tailoring hydroxyl groups of organic phenazine anodes for high-performance and stable alkaline batteries. Energy Environ. Sci. **17**(1), 114-122 (2024). <https://doi.org/10.1039/d3ee01212c>
4. Y. Liang, Y. Jing, S. Gheytani, K. Y. Lee, P. Liu et al., Universal quinone electrodes for long cycle life aqueous rechargeable batteries. Nat. Mater. **16**(8), 841-848 (2017). <https://doi.org/10.1038/nmat4919>
5. Z. Guo, J. Huang, X. Dong, Y. Xia, L. Yan et al., An organic/inorganic electrode-based hydronium-ion battery. Nat. Commun. **11**(1), 959 (2020). <https://doi.org/10.1038/s41467-020-14748-5>
6. X. Zhang, C. Fu, S. Cheng, C. Zhang, L. Zhang et al., Novel peo-based composite electrolyte for low-temperature all-solid-state lithium metal batteries enabled by interfacial cation-assistance. Energy Storage Mater. **56**, 121-131 (2023). <https://doi.org/10.1016/j.ensm.2022.12.048>
7. Q. Zhang, K. Xia, Y. Ma, Y. Lu, L. Li et al., Chaotropic anion and fast-kinetics cathode enabling low-temperature aqueous Zn batteries. ACS Energy Lett. **6**(8), 2704-2712 (2021). <https://doi.org/10.1021/acsenergylett.1c01054>
8. C. Liu, T. Ma, K. Xia, X. Hou, Q. Nian et al., High performance polyanthraquinone/co–ni(oh)2 aqueous batteries based on hydroxyl and potassium insertion/extraction reactions. Sustainable Energy Fuels **4**(1), 132-137 (2020). <https://doi.org/10.1039/c9se00598f>
9. T. Sun, C. Liu, J. Wang, Q. Nian, Y. Feng et al., A phenazine anode for high-performance aqueous rechargeable batteries in a wide temperature range. Nano Research **13**(3), 676-683 (2020). <https://doi.org/10.1007/s12274-020-2674-3>
